# Sphaerostilbellins, New Antimicrobial Aminolipopeptide Peptaibiotics from *Sphaerostilbella toxica*

**DOI:** 10.3390/biom10101371

**Published:** 2020-09-26

**Authors:** Bruno Perlatti, Connie B. Nichols, J. Andrew Alspaugh, James B. Gloer, Gerald F. Bills

**Affiliations:** 1 Texas Therapeutic Institute, The Brown Foundation Institute of Molecular Medicine, University of Texas Health Science Center at Houston, Houston, TX 77054, USA; Bruno.Perlatti@uth.tmc.edu; 2 Departments of Medicine and Molecular Genetics & Microbiology, Duke University Medical Center, Durham, NC 27710, USA; connie.nichols@duke.edu (C.B.N.); andrew.alspaugh@duke.edu (J.A.A.); 3 Department of Chemistry, University of Iowa, Iowa City, IA 52242, USA; james-gloer@uiowa.edu

**Keywords:** antifungals, Hypocreaceae, mycoparasite, nonribosomal peptide, putrescine, secondary metabolite

## Abstract

*Sphaerostilbella toxica* is a mycoparasitic fungus that can be found parasitizing wood-decay basidiomycetes in the southern USA. Organic solvent extracts of fermented strains of *S. toxica* exhibited potent antimicrobial activity, including potent growth inhibition of human pathogenic yeasts *Candida albicans* and *Cryptococcus neoformans,* the respiratory pathogenic fungus *Aspergillus fumigatus*, and the Gram-positive bacterium *Staphylococcus aureus*. Bioassay-guided separations led to the purification and structure elucidation of new peptaibiotics designated as sphaerostilbellins A and B. Their structures were established mainly by analysis of NMR and HRMS data, verification of amino acid composition by Marfey’s method, and by comparison with published data of known compounds. They incorporate intriguing structural features, including an N-terminal 2-methyl-3-oxo-tetradecanoyl (MOTDA) residue and a C-terminal putrescine residue. The minimal inhibitory concentrations for sphaerostilbellins A and B were measured as 2 μM each for *C. neoformans*, 1 μM each for *A. fumigatus*, and 4 and 2 μM, respectively, for *C. albicans*. Murine macrophage cells were unaffected at these concentrations.

## 1. Introduction

*Cryptococcus* species are among the most common causes of invasive fungal infections globally. *Cryptococcus neoformans* and *C. gattii* most often cause disease in people with compromised immune function. An estimated 220,000 cases of cryptococcosis occur annually, with mortality rates for patients with cryptococcal meningitis ranging from 10 to 70% [1,2]. As many as a third of all HIV/AIDS-associated deaths are due to cryptococcal disease, surpassing the number due to tuberculosis. Current treatments are limited to few antifungal agents (amphotericin B, flucytosine, fluconazole), with no new therapies introduced in recent decades. These therapeutics remain unsatisfactory because of their toxicity, inability to reliably eradicate the fungal pathogen and the emergence of drug resistance [3,4]. Even when treated, cryptococcosis is further complicated by high recurrence rates and the need for long-term suppressive therapy. Furthermore, the few antifungal agents currently in clinical development have not been chosen based on effectiveness against *Cryptococcus* [4,5]. As basidiomycetes, *Cryptococcus* species potentially offer unique biological perspectives to antifungal drug development compared to more broadly investigated ascomycete pathogens such as *Candida* and *Aspergillus* species. We have continued to screen metabolite-enriched fermentation extracts from unstudied ascomycete fungi and have tested these extracts in a growth-inhibition assay against the clinical strain *C. neoformans* H99 with the objective of identifying metabolites that inhibit its growth in vitro [6,7].

**Figure 1:**
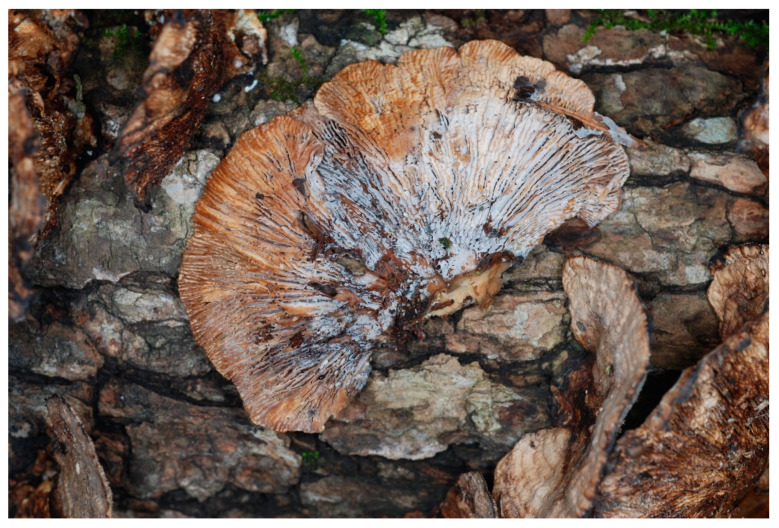
White superficial mycelium and conidia of *Sphaerostilbella toxica* parasitizing the lamellae of *Gloeophyllum striatum*. The type collection DPL-12808 (=NRRL 66954), from near Bleakwood (TX, USA), Texas. Photo by David P. Lewis.

Recently, we have cultured, fermented, and tested extracts of a new species, *Sphaerostilbella toxica,* a fungus we have found parasitizing wood-decay basidiomycetes, e.g., *Gloeophyllum striatum,* in Texas (Figure 1) and *Phellinus gilvus* in North Carolina, USA [8]. Organic solvent extracts of cultures of this mycoparasite exhibited potent antimicrobial activity, including potent growth inhibition of *C. neoformans*. *Sphaerostilbella toxica* belongs to the Hypocreaceae, a family of ascomycetes that is dominated by species that are aggressive necrotrophic or biotrophic parasites of other fungi [9]. Besides species of *Sphaerostilbella* [8], the family Hypocreaceae includes well known mycoparasitic (also known as fungicolous) genera, e.g., *Trichoderma*, *Hypomyces*, and *Escovopsis* [9,10,11]. All fungi in this family studied to date have been shown to have a complex secondary metabolism which in part contributes to their capacity to invade, kill, and colonize other fungi [12,13,14,15]. The most consistently found class of natural products of these fungi are peptaibiotics, including peptaibols, that mediate their mycoparasite life style [16,17,18,19].

Herein, we provide details on the fermentation of *S. toxica* leading to the bioactivity-guided purification and structure elucidation of new peptaibiotics with an unusual N-terminal fatty acid and C-terminal putrescine residue. Furthermore, we explored the antifungal spectrum of these new peptaibiotics.

## 2. Materials and Methods

### 2.1. Fermentation of Strains

Strains TTI-0467 (=NRRL 66953) and DPL-12808 (=NRRL 66954) of *S. toxica* from Texas, USA have been described previously [8]. Strain TTI-1079 was isolated from mycelium colonizing the basidioma of *Phellinus gilvus* collected in Raleigh, NC, USA.

To make organic extracts for antimicrobial assays, strains TTI-0467, DPL-12808, and TTI-1079 were grown on five media in 12-mL fermentations. For the seed cultures, cryopreserved agar plugs were grown for 7 days in YM agar plates (10.0 g malt extract, 2.0 g yeast extract, and 20.0 g agar in 1000 mL deionized H_2_O). Six agar discs from each culture were inoculated into 50 mL of SMY medium (40.0 g maltose, 10.0 g neopeptone, 10.0 g yeast extract, 3 g agar, 1000 mL deionized H_2_O) in a 250-mL baffled flask. Seed cultures were grown at 24 °C, 220 rpm for 4 days. One-mL aliquots of the seed cultures were transferred to 50-mL EPA glass vials containing 12 mL of the following media: for strain TTI-0467, the media set used Wheat 1 (5.0 g whole wheat seeds, 8.5 mL of base liquid consisting of yeast extract 2.0 g, sodium tartrate 10.0 g, KH_2_PO_4_ 1.0 g, MgSO_4_.7H_2_O 1.0 g, FeSO_4_.7H_2_O 0.050 g, 1000 mL deionized H_2_O), CYS80 (sucrose 80.0 g, yellow cornmeal 50.0 g, yeast extract 1.0 g, 1000 mL deionized H_2_O), MPP (maltose 25.0 g, glucose 10.0 g, dried baker’s yeast 5.0 g, Pharmamedia (Archer Daniels Midland, Decatur, IL, USA) 10.0 g, 1000 mL deionized H_2_O), YES (150.0 g sucrose, 20.0 g yeast extract, 0.5 g MgSO_4_.7H_2_O, 0.010 g ZnSO_4_.7H_2_O, 0.005 g CuSO_4_.5H_2_O, 1000 mL H_2_O) and MGP (glycerol 50.0 g, molasses 10.0 g, Pharmamedia, 5.0 g, sodium glutamate 5.0 g, NaCl 1.0 g, KH_2_PO_4_ 1.0 g, MgSO_4_.7H_2_O 0.5 g, ZnSO_4_.7H_2_O 0.05 g, 1000 mL deionized H_2_O). For strain DPL-12808, the media set used Wheat 1, CYS80, MMK2 (mannitol 40.0 g, yeast extract 5.0 g, Murashige & Skoog Salts (M-5524, Sigma Aldrich, St. Louis, MO, USA) 4.3 g, 1000 mL deionized H_2_O), Supermalt (malt extract 50.0 g, yeast extract 10.0 g, FeSO_4_.7H_2_O 0.02 g, ZnSO_4_.7H_2_O 0.007 g, 1000 mL deionized H_2_O) and MOF (mannitol 75.0 g, oat flour 15.0 g, yeast extract 5.0 g, L-glutamic acid 4.0 g, MES (2-(N-morpholino)ethanesulfonic acid) 16.2 g, 1000 mL deionized H_2_O). For strain TTI-1079, the media set used included Wheat 1, CLA (5.0 g yeast autolysate, 40.0 g corn meal, 40.0 lactose, 1000 mL deionized H_2_O), MMK2, YES and GLX (10.0 g peptone, 21.0 g malt extract, 40.0 g glycerol, 1.0 g carboxymethyl cellulose, 1000 mL deionized H_2_O). Cultures in Wheat 1, CYS80 and CLA media were incubated statically with vials slanted at a 45° angle; the other media were agitated at 220 rpm. Fermentations were grown at 24 °C for 14 days, after which 17 mL of 2-butanone was added and agitated at 220 rpm for 2 h. Eight mL of the organic phase were transferred to a clean glass vial and evaporated to dryness. The dried extracts were dissolved in 0.5 mL of DMSO prior to assay.

### 2.2. Zone of Inhibition Assays

Overnight cultures of *S. aureus* ATCC 43300 grown in Luria-Bertani medium (LB), Sigma Aldrich), and *C. albicans* ATCC 10231 and *C. neoformans* H99 grown in YM broth (malt extract 10 g, yeast extract 2 g in deionized 1000 mL H_2_O) at 37 °C were diluted with sterile H_2_O to an OD_600_ of 0.4 for *S. aureus* and 0.8 for *C. albicans* and *C. neoformans*. One-milliliter aliquots of the resulting cell suspensions were mixed with 35 mL aliquots of YMA (LB agar for *S. aureus*) at 45 °C and dispensed into one-well Omnitray (Thermofisher Nunc, Grand Island, NY, USA) plates. A 35-well pattern of test wells (4 mm diam) was formed in the solidified agar by aspiration of wells with a Luer-lock syringe or by molding wells with a custom 3D-printed pin lid (V & P Scientific, San Diego, CA, USA), and 10 µL of each extract in DMSO was applied to each well. *C. albicans* was incubated at 25 °C, *S. aureus* at 37 °C and *C. neoformans* was incubated at both temperatures. Zones of inhibition were photographed after 72 h. During screening, amphotericin B was the positive control or antifungal assays and a mixture of chlortetracycline and streptomycin were the positive controls for antibacterial assays.

### 2.3. Isolation and Characterization of Active Compounds from Strain TTI-0467

Strain TTI-0467 was selected for scale up and isolation of the active compounds. Bioassay-guided fractionation was used to identify active compounds in the crude extract of TTI-0467. The strain was grown in1-L bottles containing YES liquid media infused into vermiculite as a solid matrix to promote surface growth and mycelial differentiation [20]. To prepare the media, 340 mL of coarse vermiculite were added to each of eight 1-L glass bottles and autoclaved. When bottles were cooled, 120 mL of liquid YES media were added to each bottle along with 2 mL of a four-day-old TTI-0467 seed culture grown on SMYA liquid medium. Bottles were rotated on a cell production roller apparatus (Bellco, Vineland, NJ, USA,) until the mycelium formed a solid mass in the vermiculite matrix after 3 days, then bottles continued incubating without rolling. After 14 days, 350 mL of 2-butanone was added to each 1-L bottle and agitated for 4 h at 220 rpm. The extracted material was coarsely filtered to remove mycelium and vermiculite, and then filtered a second time to remove additional mycelia. The organic phase was separated and dried under vacuum, yielding 1.89 g of crude extract. The extract was adsorbed onto Celite (diatomaceous earth) and loaded into a vacuum liquid chromatography column (silica gel 60, 10 × 7 cm). Fractions were eluted successively with hexane, ethyl acetate (EtOAc):methanol (MeOH) (1:1 *v*/*v*), and MeOH. The MeOH fraction was dried, applied to a LH-20 column (30 × 3 cm, Sephadex, GE Healthcare, Uppsala, Sweden) and eluted with MeOH:CH_2_Cl_2_ (1:1). Fractions 6–15 containing the bioactive compounds were pooled and dried. The resulting mixture was purified using semi-preparative HPLC (Zorbax SB-C_18_ column, Agilent Technologies, Santa Clara, CA, USA); 5 μm; 9.4 × 250 mm, 40 °C; isocratic ternary mixture for 25 min (40.5:19.0:40.5 A:B:C; (A), 0.1% trifluoroacetic acid (TFA) in acetonitrile, (B), 0.1% TFA in H_2_O; solvent C, MeOH, 4.0 mL/min), yielding 70 mg of **1** (sum of the two isomers) and 36 mg of **2** (sum of the two isomers). Peak identity was confirmed by HPLC (Ace Equivalence C_18_ column 150 × 4.6 mm, 5-µm, 30 °C). Gradient elution, 60% A for 6 min then from 60–70% A in 12 min, 1.0 mL/min. The mobile phase consisted of (A) 0.1% formic acid in acetonitrile and (B) 0.1% aqueous formic acid. 

NMR data were collected on a 500-MHz NMR instrument (Bruker, Billerica, MA, USA) equipped with a 5-mm triple resonance cryoprobe at 298 K, with DMSO-*d*_6_ as solvent. Semipreparative separations and HPLC-MS analyses were performed on an Agilent HPLC 1260 system equipped with a diode array detector (DAD) and coupled to an Agilent 6120 single quadrupole mass spectrometer (MS). HRMS data were acquired on an Orbitrap Fusion^TM^ Tribrid^TM^ mass spectrometer (Thermo Scientific^TM^, Grand Island, NY, USA) with direct infusion. The Orbitrap Fusion was operated with measurement of FTMS^1^ at a resolution of 120,000 FWHM, scan range of *m*/*z* 150–1250, automated gain control (ACG) target 2e5, and maximum injection time of 100 ms; the FTMS^2^ spectra were collected with optimum higher collisional dissociation (HCD) energy, scan range *m*/*z* 150–2000, AGC target 5e4, 2 *m*/*z* isolation window.

### 2.4. Determination of Absolute Configuration of Amino Acids

The determination of the absolute configuration of the amino acid units in **1** and **2** was accomplished using a modified version of Marfey’s method [21]. The HPLC analysis used the same column used for analysis of fermentation extracts and was held at 30 °C. To achieve resolution between β-Ala and L-Ala, the same ternary solvent mixture used for semi-preparative separation was applied in a gradient from 10:80:10 to 20:60:20 A:B:C in 28 min, and then to 50:0:50 during 12 more min, totaling 40 min of analysis, at 1.0 mL/min. Amino acid derivatives were detected by UV at 340 nm and by positive ion electrospray ionization mass spectrometry (ESI-MS).

### 2.5. Minimum Inhibitory Concentration (MIC) Assays 

To quantify the inhibitory concentrations of the sphaerostilbellins for select strains of fungal and bacterial pathogens, MICs were measured using species-specific modifications to standard CLSI testing methods [22]. Overnight cultures of *S. aureus* ATCC 43300 in LB media, and *C. albicans* ATCC 10231 and *C. neoformans* H99 in YM media were diluted with sterile H_2_O to an OD_600_ of 0.4 for *S. aureus* and 0.8 for *C. albicans* and *C. neoformans*. For *Aspergillus fumigatus* FGSC A1240, conidia from a sporulating agar culture were used. Each suspension of cells or conidia was diluted 1000× in RPMI-1640 buffered with MOPS (Sigma-Aldrich). Stock solutions of the active compounds were prepared in DMSO at 1.28 mM. The final cell suspension (195 µL) was transferred to a 96-well plate containing 5 µL of stock solution for each active compound, and subsequently serially diluted to achieve a dose range of 0.06–64 µM. Growth was assessed at 24 h (*S. aureus* and *C. albicans*) or 72 h (*C. neoformans, A. fumigatus*) by adding 10% alamarBlue (Bio-Rad, Hercules, CA, USA) and incubating in the dark at 37 °C until color developed. Each compound was tested in duplicate. A streptomycin-chlortetracycline mixture was used as a positive control for *S. aureus*, and amphotericin B was used for *C. albicans* and *C. neoformans*.

### 2.6. Macrophage-Fungal Co-Incubation and Macrophage Cytotoxicity Assays

The macrophage-*C. neoformans* co-incubation assay was adapted from [23]. J774A.1 macrophage-like murine cells grown in DMEM (Dulbecco’s Modified Eagle Medium, Sigma-Aldrich) were harvested, washed in PBS, transferred to 96-well tissue culture plates at 10^5^ cells/well, and incubated overnight at 37 °C and 5% CO_2_. The *C. neoformans* strain H99 was incubated in YPD medium (1% yeast extract, 2% peptone, 2% dextrose) overnight with shaking at 37 °C, washed with PBS, and resuspended in DMEM medium at 10^3^ cells/ml. Sphaerostilbellins (**1** and **2**) were prepared in serial 2-fold dilutions in DMEM and DMEM containing H99 cells to achieve a dose range of 0.03–32 µM. The macrophage medium was replaced with the DMEM medium containing compound or fungal cells plus compound and incubated for 24 to 48 h at 37 °C and 5% CO_2_. Macrophage viability was assessed by the addition of 10% alamarBlue and incubating for 3 h at 37 °C and 5% CO_2_ prior to fluorescence measurement (FLUORStar Optima plate reader, BMG Labtech, Cary, NC, USA). Fungal growth was assessed visually at 48 h. To test for fungal survival, samples from each well were plated onto YPD agar and incubated at 30 °C. Each compound was tested in duplicate biological replicates.

## 3. Results and Discussion

During screening of extracts from fungal fermentations for antifungal activity using agar zone of inhibition assays, extracts of *S. toxica*, TTI-0467, DPL-12808, and TTI-1079, exhibited large, clear inhibition zones against *Staphylococcus aureus*, *Candida albicans* and *C. neoformans* [8] on most of the media tested (Figures S1–S3). 

The bioactive components were tracked by bioactivity-guided HPLC fractionation of the crude extract of strain TTI-0467 grown in a solid wheat medium. HPLC analysis indicated the presence of at least two active compounds. Chromatographic separations afforded an enriched fraction containing four peaks, from which peaks 1 (minor) and 3 (major) showed *m*/*z* = 1701 and peaks 2 (minor) and 4 (major) had *m*/*z* = 1786. The high molecular weight and the absence of UV-visible absorption suggested a peptidic nature for the molecules. Upon separation of all four peaks and subsequent HPLC analysis, it was observed that upon reinjection of individual peaks, both pairs of peaks with the same nominal mass interconverted between two forms, with the predominance of peaks 3 and 4 (Figure S4). While NMR analysis of purified compounds in CD_3_OD showed a complex superposition of two different sets of signals (Figure S5), spectra obtained for molecules in DMSO-*d6* coalesced to a single set of sharp signals. Therefore, for structure elucidation, peaks 2 and 4, representing the major compound, were considered as compound **1**, while peaks 1 and 3 were defined as compound **2**.

Initial HRMS analysis determined the molecular formula of **1** as C_86_H_152_N_20_O_20_ (*m/z* 893.5828, [M + 2H]^2+^, Δ = 0.40 ppm), and for compound **2** as C_82_H_145_N_19_O_19_ (*m/z* 851.0560, [M + 2H]^2+^, Δ = −0.03 ppm). The 85.0528 Da difference between the two molecules corresponded to a C_4_H_7_NO residue indicating that **2** had one less aminoisobutyric acid (Aib) residue. Comparison of HRMS data with peptide databases [17,24,25] identified two lipopeptaibiotics, SCH 466,457 and SCH 466,456 from an unidentified fungus characterized at Schering Plough [26] that had the same molecular weights as **1** and **2,** respectively. However, ^1^H- and ^13^C-NMR analysis for **1** and **2** (Table 1), revealed structural differences between the isolated compounds and the SCH peptaibols. The most notable differences were the absence of a ^13^C-NMR peak at δ 158, indicating the lack of an arginine residue, and the presence of several correlated multiplets at δ 2.2–2.4 and δ 3.0–3.3 in the ^1^H-NMR spectra, indicating the presence of β-amino acids, whereas in the SCH peptaibols only a glycine peak was observed in this region at δ 3.05. Moreover, the earlier report made no mention of an interconversion between two forms. These differences led us to undertake a thorough characterization of the chemical structures of **1** and **2**.

**Figure 2:**
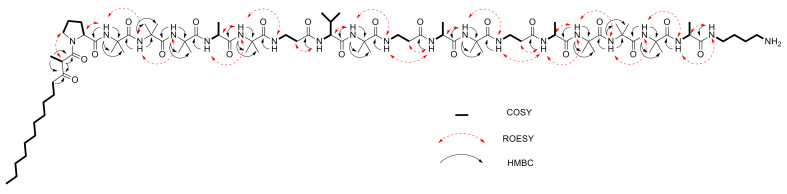
2D NMR correlations used for structural characterization of compound **1**.

For the major compound, **1**, ^1^H-, ^13^C-, and 2D NMR experiments (COSY, HSQC, HMBC, ROESY) revealed the presence of nine 2-aminoisobutyric acid (Aib) residues, as well as four alanine (Ala) units, three β-alanine (β-Ala) units, one valine (Val) unit, and one proline (Pro) unit (Table 1, Figures S6–S11). Linked to the proline residue at the N-terminus was a 2-methyl-3-oxotetradecanoyl (MOTDA) residue (Figure 2). Observation of a ROESY correlation between the α-methine hydrogen of the MOTDA residue with Pro2 δH indicated a *s-trans* configuration of the Pro residue (Table 1, Figure 2). Furthermore, COSY correlations also showed a spin system arising from an amide NH proton at δ 7.39 connected to a 4-aminobutyl side-chain. The terminal H-4 methylene protons at δ 2.78 showed a COSY correlation with an NH_2_ proton signal at δ 7.69, characterized by a surprisingly strong deshielding effect, similar to observations in other peptaibiotics [27]. Extensive analysis of HMBC and ROESY data guided determination the amino acid sequence and the locations of the other units (Table 2, Figure 2).

**Figure 3:**
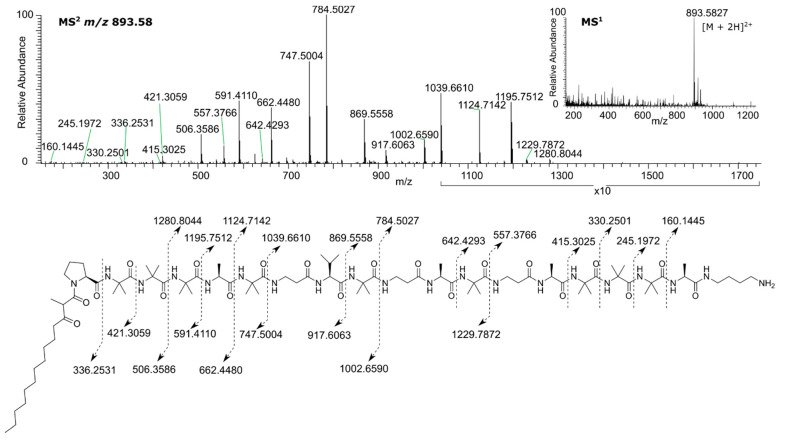
HRMS for sphaerostilbellin A (**1**).

The assigned sequence of amino acids was supported by HRMS/MS analysis (Figure 3). Fragmentation of the [M + 2H]^2+^ ion at *m*/*z* 893.5827 resulted in a series of observable b/y fragments, in accordance with the sequential loss of amino acid residues from the N- and C- termini, respectively. Interestingly, formation of specific pairs of b/y fragments derived from the α-cleavage of β-Ala ions were greatly suppressed or absent, a feature common in other peptaibiotics containing β-Ala residues [28,29], which further supported the existence and positions of the β-Ala units in the proposed amino acid sequence.

**Figure 4:**
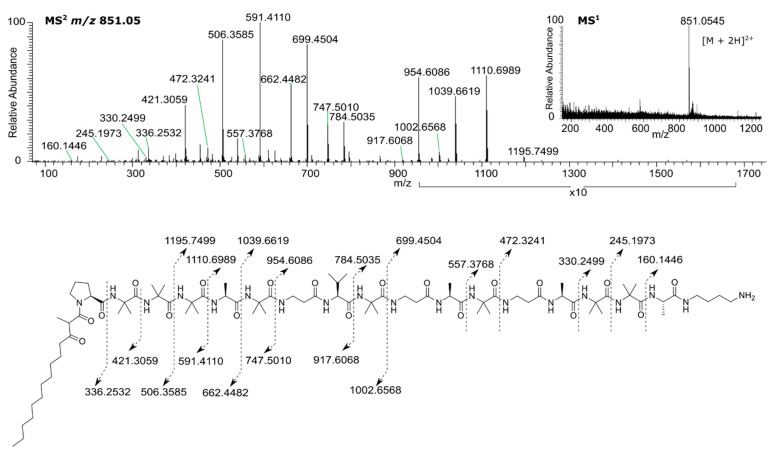
HRMS for sphaerostilbellin B (**2**).

**Figure 5:**
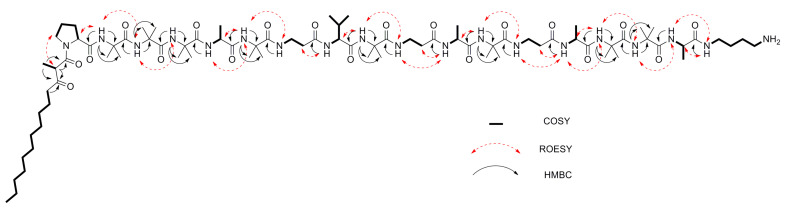
2D NMR correlations used for structural characterization of compound **2**.

MS^2^ analysis of the ion at *m*/*z* 851.0560 corresponding to the [M + 2H]^2+^ ion for **2** showed an identical b ion pattern and a y ion series containing the incremental mass difference between **1** and **2**, indicating the missing Aib among the last sequence of Aib residues (Figure 4). Analysis of ^1^H NMR data showed the absence of an NH amide proton at δ 7.80, attributed to residue Aib_16_ in **1**. The absence of this peak, and the observation of a ROESY correlation between the amide NH proton of Aib_15_ (δ 8.41) with δ7.70, attributed to the NH amide proton of residue Aib_17_ in **1** confirmed the absence of the residue Aib_16_ in **2**. NMR data (Figure 5, Figures S12–S17) for **2** are provided in Table 2. 

Marfey’s analysis of the amino acids present in the acid hydrolyzates of **1** and **2** confirmed the absolute configurations of the chiral amino acids as L-Ala, L-Pro, and L-Val (Figure S18). The LC-MS analysis of the Marfey’s derivative standard amino acid mixture also confirmed the presence of β-Ala, Aib, and putrescine. L-Arg and Gly, present in the SCH peptaibols, were included among the derivatized standards for comparison but were absent from the samples, further confirming the differences among these compounds. It should be noted that relatively low absorbance for Aib and putrescine derivatives evident in chromatograms (Figure S18) are likely due to slower derivatization reaction rates required for complete reaction with Marfey’s reagent [30,31].

The sphaerostilbellins exhibit several distinctive features. The presence of a primary amine unit at the C-terminal end is rare and only found in a few examples of peptaibiotics such as cicadapeptins [27] and MS-681a-d, isolated from a *Myrothecium* species [32]. To the best of our knowledge, this is the first report of a putrescine residue in a fungus-derived peptaibiotic. Substitution at the N-terminus with the polyketide-derived MOTDA unit has been reported in only a few other peptaibiotics such as lipohexin [33,34], SCH 466,456 and SCH466457 [26], SCH 643,432 and its unidentified isomer [35], and texenomycins A and B [36]. In the latter two examples, the pair of isolated peptaibiotics were characterized as isomers, and in the texenomycins, they differed in the stereochemistry of the methyl group arranged between the two carbonyl groups of the MOTDA residue. 

In order to investigate whether the isomerization of sphaerostilbellins in LC-MS chromatograms was due to methyl group isomerization through a keto-enol equilibrium at the β-keto amide methine, a sample of purified sphaerostilbellin A was dissolved in CD_3_OD and left at room temperature for an extended period of time. Even after 110 days exposed to the protic solvent, there was no noticeable change in the ^1^H-NMR signal attributed to the methine hydrogen (Figure S19), indicating no appreciable deuterium exchange at the methine hydrogen and thus no racemization at this position under these conditions. Another possible explanation for the presence of two forms in CD_3_OD solution could arise from *cis*-*trans* proline conformational isomerization, a slow-exchange phenomenon that is widely recognized and observed in peptides, especially when proline is located as the penultimate residue [37,38,39]. ROESY analysis of 1 in CD_3_OD was consistent with the major conformer adopting a *s-trans* conformation, similar to what was observed in DMSO-*d6*, however, these data did not provide positive evidence to unambiguously identify the minor form as the *s-cis* conformer. 

Minimum inhibitory concentration (MIC) assays in liquid media for compounds **1** and **2** indicated strong potency towards all tested pathogens; *S. aureus, A. fumigatus*, *C. albicans*, and *C. neoformans*, with *A. fumigatus* exhibiting the highest sensitivity (Table 3). Antifungal activity was observed at lower concentrations than for inhibition *of S. aureus* for both compounds. Compound **1** showed a consistent two to four times lower MIC when compared to compound **2**. Compounds **1** and **2** were also tested for toxicity against the murine macrophage-like cell line J774A.1 This cell line has been used extensively to assess in vitro phagocytic and antifungal activity against *C. neoformans* and other fungal pathogens [23,40]. Each compound displayed much higher inhibition/toxicity against these mammalian cells compared to fungal and bacterial cells, potentially arguing against their acting as non-specific cell toxins (Table 3). Additionally, the antifungal MIC for *C. neoformans* was identical whether the compounds were assayed with fungal cells alone or in a macrophage-fungal co-culture.

## 4. Conclusions

Strong antibacterial and antifungal activity and cell lysis are typical biological effects of lipopeptaibiotics [41,42]. In most cases, their biological activities are believed to be due to their membrane-modifying properties and ability to form transmembrane voltage-dependent channels or to cause destabilization or leakage of membrane lipid bilayers. Therefore, production of sphaerostibellins by *S. toxica* is consistent with the chemical behavior of other mycoparasitic fungi of the Hypocreaceae. The incorporation of a C-terminal putrescine residue, an intermediate in fungal polyamine metabolism [43], represents a new variation in the family. Although the sphaerostilbellins displayed broad biological toxicity, their effect was more pronounced on the fungi tested, which might be correlated with their ecological role, and warrants further investigation of the precise mechanism of their antifungal activity. In conclusion, the strategy of screening novel fungal species of mycoparasitic Hypocreaceae [19], e.g., *S. toxica*, has led to the discovery of two novel and potent antimicrobial peptaibiotics with the primary amine putrescine at the C-terminal end.

## Figures and Tables

**Table 1:** ^1^H- and ^13^C-NMR data for compound **1** in DMSO (500 and 125 MHz for ^1^H- and ^13^C-, respectively).

Residue.	Position	δC	δH, mult (J in Hz)	Residue	Position	δC	δH, mult (J in Hz)
MOTDA	1	169.40		Aib_9_	NH		8.02, s
	2	50.83	3.80, q (6.9)		α	55.85	
	2’	12.45	1.15, d (6.9)		β-1	23.13	1.39, m ^a^
	3	206.39			β-2	24.30	1.33, m ^a^
	4	40.42	2.47, m		C=O	174.08	
	5	22.84	1.40, m	β-Ala_10_	NH		7.44, t (5.5)
	6	22.04	1.22, m		α	35.81	a: 3.32, m; b: 3.14, m
	7	28.69	1.16, m		β	35.09	2.29, m
	8	28.89	1.20, m		C=O	171.18	
	9	28.95	1.21, m	L-Ala_11_	NH		8.12, d (4.8)
	10	28.82	1.24, m		α	49.19	4.10, q (6.6)
	11	28.99	1.25, m		β	17.09	1.18, m
	12	28.64	1.36, m		C=O	172.20	
	13	31.23	1.23, m	Aib_12_	NH		8.04, s
	14	13.91	0.85, m		α	56.06	
L-Pro_1_	α	60.13	4.26, dd (5.8, 7.8)		β-1	23.38	1.27, m ^a^
	β	28.45	a: 2.16, m; b: 1.93, m		β-2	25.75	1.37, m ^a^
	γ	24.57	a: 2.02, m; b: 1.92, m		C=O	174.09	
	δ	47.57	3.64, t (6.6)	β-Ala_13_	NH		7.50, t (5.6)
	C=O	172.43			α	35.83	a: 3.30, m; b: 3.21, m
Aib_2_	NH		8.55, s		β	35.06	2.31, m
	α	55.96			C=O	171.54	
	β1	23.90	1.34, m ^a^	L-Ala_14_	NH		8.19, d (4.7)
	β2	25.50	1.32, m ^a^		α	49.94	4.03, m ^d^
	C=O	175.38			β	16.63	1.24, m
Aib_3_	NH		7.95, s		C=O	174.01	
	α	55.84		Aib_15_	NH		8.40, s
	β-1	24.58	1.39, m ^a^		α	55.99	
	β-2	25.74	1.36, m ^a^		β-1	24.10	1.37, m ^a^
	C=O	175.19			β-2	24.96	1.36, m ^a^
Aib_4_	NH		7.70, s ^b^		C=O	175.34	
	α	55.88		Aib_16_	NH		7.80, s
	β-1	24.92	1.34, m ^a^		α	55.69	
	β-2	26.46	1.30, m ^a^		β-1	22.93	1.29, m ^a^
	C=O	175.14			β-2	25.80	1.32, m ^a^
L-Ala_5_	NH		7.71, d ^b^		C=O	175.26	
	α	50.02	3.93, m ^c^	Aib_17_	NH		7.67, s
	β	16.47	1.28, m		α	55.95	
	C=O	172.00			β-1	21.99	1.33, m ^a^
Aib_6_	NH		7.34, s		β-2	26.64	1.35, m ^a^
	α	56.01			C=O	173.37	
	β-1	24.67	1.36, m ^a^	L-Ala_18_	NH		7.56, d (7.8)
	β-2	24.70	1.36, m ^a^		α	48.96	4.04, m ^d^
	C=O	174.02			β	17.11	1.31, m
β-Ala_7_	NH		7.13, t (5.4)		C=O	172.05	
	α	35.60	a: 3.28, m; b: 3.18, m	Put	NH		7.39, t (5.6)
	β	35.20	2.35, m		α	37.90	a: 3.10, m; b: 3.02, m
	C=O	171.04			β	25.88	1.50, m ^e^
L-Val_8_	NH		8.00, d (7.0)		γ	24.26	1.53, m ^e^
	α	58.93	3.92, m ^c^		δ	38.53	2.78, m
	β	29.58	1.93, m		NH2		7.69, m ^b^
	γ-1	19.08	0.86, m				
	γ-2	18.68	0.87, m				
	C=O	170.78					

^a–f^ Signals are overlapped. MOTDA - 2-methyl-3-oxo-tetradecanoyl.

**Table 2:** ^1^H- and ^13^C-NMR data for compound **2** in DMSO-*d*_6_ (500 and 125 MHz for ^1^H- and ^13^C-, respectively).

Residue	Position	δC	δH, mult (J in Hz)	Residue	Position	δC	δH, mult (J in Hz)
MOTDA	1	169.40		Aib_9_	NH		8.02, s
	2	50.83	3.80, q (6.9)		α	55.85	
	2’	12.45	1.15, d (6.9)		β-1	23.13	1.39, m ^a^
	3	206.39			β-2	24.30	1.33, m ^a^
	4	40.42	2.47, m		C=O	174.08	
	5	22.84	1.40, m ^a^	β-Ala_10_	NH		7.44, m ^d^
	6	22.04	1.24, m ^f^		α	35.81	a: 3.32, m; b: 3.14, m
	7	28.69	1.16, m ^f^		β	35.09	2.29, m
	8	28.89	1.20, m ^f^		C=O	171.18	
	9	28.95	1.21, m ^f^	L-Ala_11_	NH		8.10, d (4.8)
	10	28.82	1.24, m ^f^		α	49.19	4.10, q (6.6)
	11	28.99	1.25, m ^f^		β	17.15	1.18, m
	12	28.64	1.36, m ^a^		C=O	172.20	
	13	31.23	1.23, m ^f^	Aib_12_	NH		8.04, s
	14	13.91	0.85, m		α	56.06	
L-Pro_1_	α	60.13	4.26, dd (5.8, 7.8)		β-1	23.38	1.27, m ^a^
	β	28.45	a: 2.16, m; b: 1.93, m		β-2	25.75	1.37, m ^a^
	γ	24.57	a: 2.02, m; b: 1.92, m		C=O	174.03	
	δ	47.57	3.64, t (6.4) m	β-Ala_13_	NH		7.50, m ^e^
	C=O	172.43			α	35.83	a: 3.30, m; b: 3.21, m
Aib_2_	NH		8.55, s		β	35.06	2.31, m
	α	55.96			C=O	171.54	
	β1	23.90	1.34, m ^a^	L-Ala_14_	NH		8.14, d (4.7)
	β2	25.50	1.32, m ^a^		α	49.70	4.03, m ^g^
	C=O	175.38			β	16.74	1.24, m
Aib_3_	NH		7.95, s		C=O	173.97	
	α	55.84		Aib_15_	NH		8.41, s
	β-1	24.58	1.39, m ^a^		α	55.99	
	β-2	25.74	1.36, m ^a^		β-1	23.90	1.37, m ^a^
	C=O	175.19			β-2	25.27	1.36, m ^a^
Aib_4_	NH		7.70, s ^b^		C=O	175.00	
	α	55.88		Aib_16_	NH		7.71, s
	β-1	24.92	1.34, m ^a^		α	55.86	
	β-2	26.46	1.30, m ^a^		β-1	21.99	1.33, m ^a^
	C=O	175.14			β-2	26.47	1.35, m ^a^
L-Ala_5_	NH		7.71, d^b^		C=O	173.49	
	α	50.02	3.93, m ^c^	L-Ala_17_	NH		7.49, m ^e^
	β	16.47	1.28, m		α	48.96	4.04, m ^g^
	C=O	172.00			β	17.27	1.31, m
Aib_6_	NH		7.34, s		C=O	172.05	
	α	56.01		Put	NH		7.45, m ^d^
	β-1	24.67	1.36, m ^a^		α	37.9	a: 3.10, m; b: 3.02, m
	β-2	24.70	1.36, m ^a^		β	25.88	1.50, m ^h^
	C=O	174.02			γ	24.26	1.53, m ^h^
β-Ala_7_	NH		7.13, t (5.4)		δ	38.49	2.78, m
	α	35.60	a: 3.28, m; b: 3.18, m		NH2		7.69, m ^b^
	β	35.20	2.35, m				
	C=O	171.04					
L-Val_8_	NH		8.00, d (7.0)				
	α	58.93	3.92, m ^c^				
	β	29.58	1.93, m				
	γ-1	19.08	0.86, m				
	γ-2	18.68	0.87, m				
	C=O	170.78					

^a–e^ Signals are overlapped. MOTDA - 2-methyl-3-oxo-tetradecanoyl.

**Table 3:** Minimum inhibitory concentration (MIC) values of peptaibiotics 1 and 2 against selected pathogens and murine macrophage cell line J774A.1.

Pathogen or Cell Line	Compound (μM)	
	Sphaerostilbellin A (1)	Sphaerostilbellin B (2)
*C. neoformans* H99 37 °C	2	2
*C. neoformans* H99 30 °C	2	2
*C. albicans* ATCC 10231	4	2
*A. fumigatus* FGSC A1240	1	1
*S. aureus* ATCC 43300	8	32
Murine macrophage J774A.1	>32	32

## Supplementary Materials

The following are available online at https://www.mdpi.com/2218-273X/10/10/1371/s1, Figure S1: Zone of inhibition assay of extracts from strain TTI-0467 cultured in five different media against *C. neoformans* at 37 °C. See Methods for media formulations; Figure S2. Zone of inhibition assay of extracts from strain DPL-12808 cultured in five different media against *C. neoformans* at 37 °C. See Methods for media formulations; Figure S3. Zone of inhibition assay of extracts of strain TTI-1079 cultured in five different media against *C. neoformans* at 37 °C. See Methods for media formulations; Figure S4. LC-MS of peaks 1-4 after semi-preparative HPLC isolation, highlighting the interconversion of peaks 1-3 and 2-4; Figure S5. ^1^H NMR spectrum of peaks 1-4 (500 MHz, CD_3_OD); Figure S6. ^1^H NMR spectrum of 1 (500 MHz, DMSO-*d_6_*); Figure S7. ^13^C NMR spectrum of 1 (125 MHz, DMSO-*d_6_*); Figure S8. COSY spectrum of 1 (500 MHz, DMSO-*d_6_*); Figure S9. HSQC spectrum of 1 (500 MHz, DMSO-*d_6_*); Figure S10. HMBC spectrum of 1 (500 MHz, DMSO-*d_6_*); Figure S11. ROESY spectrum of 1 (500 MHz, DMSO-*d_6_*); Figure S12. ^1^H NMR spectrum of 2 (500 MHz, DMSO-*d_6_*); Figure S13. ^13^C NMR spectrum of 2 (125 MHz, DMSO-*d_6_*); Figure S14. COSY spectrum of 2 (500 MHz, DMSO-*d_6_*); Figure S15. HSQC spectrum of 2 (500 MHz, DMSO-*d_6_*); Figure S16. HMBC spectrum of 2 (500 MHz, DMSO-*d_6_*); Figure S17. ROESY spectrum of 2 (500 MHz, DMSO-*d_6_*); Figure S18. LCMS chromatograms obtained after sample derivatization with Marfey’s reagent. (a) Putrescine standard; (b) Amino acid standards; (c) Compound 1 hydrolyzate; (d) Compound 2 hydrolyzate. X represents a side-product; Figure S19. ^1^H NMR (500 MHz, CD_3_OD) spectra of sphaerostilbellin A at different times. (a) 0 days; (b) 4 days; (c) 110 days.

## References

[1] Benedict K., Jackson B.R., Chiller T., Beer K.D. (2018). Estimation of direct healthcare costs of fungal diseases in the United States. Clin. Infect. Dis..

[2] Rajasingham R., Smith R.M., Park B.J., Jarvis J.N., Govender N.P., Chiller T.M., Denning D.W., Loyse A., Boulware D.R. (2017). Global burden of disease of HIV-associated cryptococcal meningitis: An updated analysis. Lancet Infect. Dis..

[3] Brown G.D., Denning D.W., Gow N.A.R., Levitz S.M., Netea M.G., White T.C. (2012). Hidden killers: Human fungal infections. Sci. Transl. Med..

[4] Perfect J.R. (2017). The antifungal pipeline: A reality check. Nat. Rev. Drug Discov..

[5] Van Daele R., Spriet I., Wauters J., Maertens J., Mercier T., Van Hecke S., Bruggemann R. (2019). Antifungal drugs: What brings the future?. Med. Mycol..

[6] Xu L., Li Y., Biggins J.B., Bowman B.R., Verdine G.L., Gloer J.B., Alspaugh J.A., Bills G.F. (2018). Identification of cyclosporin C from *Amphichorda felina* using a *Cryptococcus neoformans* differential temperature sensitivity assay. Appl. Microbiol. Biotechnol..

[7] Li Y., Yue Q., Jayanetti D.R., Swenson D.C., Bartholomeusz G.A., An Z., Gloer J.B., Bills G.F. (2017). Anti-cryptococcus phenalenones and cyclic tetrapeptides from *Auxarthron pseudauxarthron*. J. Nat. Prod..

[8] Põldmaa K., Bills G., Lewis D.P., Tamm H. (2019). Taxonomy of the *Sphaerostilbella broomeana*-group (Hypocreales, Ascomycota). Mycol. Prog..

[9] Gams W., Diederich P., Põldmaa K., Mueller G.M., Bills G.F., Foster M.S. (2004). Chapter 17 - Fungicolous fungi. Biodiversity of Fungi.

[10] Rossman A.Y., Samuels G.J., Rogerson C.T., Lowen R. (1999). Genera of Bionectriaceae, Hypocreaceae and Nectriaceae (Hypocreales, Ascomycetes). Stud. Mycol..

[11] Kredics L., Hatvani L., Naeimi S., Körmöczi P., Manczinger L., Vágvölgyi C., Druzhinina I., Gupta V.K., Schmoll M., Herrera-Estrella A., Upadhyay R.S., Druzhinina I., Tuohy M.G. (2014). Chapter 1 - Biodiversity of the genus Hypocrea/Trichoderma in different habitats. Biotechnology and Biology of Trichoderma.

[12] Dhodary B., Schilg M., Wirth R., Spiteller D. (2018). Secondary metabolites from *Escovopsis weberi* and their role in attacking the garden fungus of leaf-cutting ants. Chem. Eur. J..

[13] Nair M.S.R., Carey S.T., James J.C. (1981). Metabolites of pyrenomycetes. XIV. Structure and partial stereochemistry of the antibiotic macrolides hypothemycin and dihydrohypothemycin. Tetrahedron.

[14] Röhrich C.R., Jaklitsch W.M., Voglmayr H., Iversen A., Vilcinskas A., Nielsen K.F., Thrane U., von Döhren H., Brückner H., Degenkolb T. (2014). Front line defenders of the ecological niche! Screening the structural diversity of peptaibiotics from saprotrophic and fungicolous *Trichoderma/Hypocrea* species. Fungal Divers..

[15] Li D., Sossah F.L., Sun L., Fu Y., Li Y. (2019). Genome analysis of *Hypomyces perniciosus,* the causal agent of wet bubble disease of button mushroom (*Agaricus bisporus*). Genes.

[16] Degenkolb T., Kirschbaum J., Brückner H. (2007). New sequences, constituents, and producers of peptaibiotics: An updated review. Chem. Biodivers..

[17] Neumann N.K.N., Stoppacher N., Zeilinger S., Degenkolb T., Brückner H., Schuhmacher R. (2015). The peptaibiotics database—A comprehensive online resource. Chem. Biodivers..

[18] Degenkolb T., von Döhren H., Fog Nielsen K., Samuels G.J., Brückner H. (2008). Recent advances and future prospects in peptaibiotics, hydrophobin, and mycotoxin sesearch, and their importance for chemotaxonomy of *Trichoderma* and *Hypocrea*. Chem. Biodivers..

[19] Degenkolb T., Berg A., Gams W., Schlegel B., Grafe U. (2003). The occurrence of peptaibols and structurally related peptaibiotics in fungi and their mass spectrometric identification via diagnostic fragment ions. J. Pept. Sci..

[20] Bills G.F., Dombrowski A.W., Goetz M.A. (2012). The "FERMEX" method for metabolite-enriched fungal extracts. Meth. Mol. Biol..

[21] Ayers S., Ehrmann B.M., Adcock A.F., Kroll D.J., Carcache de Blanco E.J., Shen Q., Swanson S.M., Falkinham III J.O., Wani M.C., Mitchell S.M. (2012). Peptaibols from two unidentified fungi of the order Hypocreales with cytotoxic, antibiotic, and anthelmintic activities. J. Pept. Sci..

[22] Alexander B.D. (2017). Reference Method for Broth Dilution Antifungal Susceptibility Testing of Yeasts. Approved Standard M27–A4.

[23] Samantaray S., Correia J.N., Garelnabi M., Voelz K., May R.C., Hall R.A. (2016). Novel cell-based in vitro screen to identify small-molecule inhibitors against intracellular replication of *Cryptococcus neoformans* in macrophages. Int. J. Antimicrobiol. Agents.

[24] Flissi A., Ricart E., Campart C., Chevalier M., Dufresne Y., Michalik J., Jacques P., Flahaut C., Lisacek F., Leclère V. (2019). Norine: Update of the nonribosomal peptide resource. Nucl. Acid. Res..

[25] Whitmore L., Wallace B.A. (2004). The peptaibol database: A database for sequences and structures of naturally occurring peptaibols. Nucl. Acid. Res..

[26] Hegde V.R., Silver J., Patel M., Gullo V.P., Yarborough R., Huang E., Das P.R., Puar M.S., DiDomenico B.J., Loebenberg D. (2001). Novel fungal metabolites as cell wall active antifungals: Fermentation, isolation, physico-chemical properties, structure and biological activity. J. Antibiotics.

[27] Krasnoff S.B., Reátegui R.F., Wagenaar M.M., Gloer J.B., Gibson D.M. (2005). Cicadapeptins I and II:  New Aib-containing peptides from the entomopathogenic fungus *Cordyceps heteropoda*. J. Nat. Prod..

[28] Brückner H., Fox S., Degenkolb T. (2019). Sequences of acretocins, peptaibiotics containing the rare 1-aminocyclopropanecarboxylic Aacid, from *Acremonium crotocinigenum* CBS 217.70. Chem. Biodivers..

[29] Du L., Risinger A.L., Mitchell C.A., You J., Stamps B.W., Pan N., King J.B., Bopassa J.C., Judge S.I.V., Yang Z. (2017). Unique amalgamation of primary and secondary structural elements transform peptaibols into potent bioactive cell-penetrating peptides. Proc. Nat. Acad. Sci. USA.

[30] Vemula H., Kitase Y., Ayon N.J., Bonewald L., Gutheil W.G. (2017). Gaussian and linear deconvolution of LC-MS/MS chromatograms of the eight aminobutyric acid isomers. Anal. Biochem..

[31] Ayon N.J., Sharma A.D., Gutheil W.G. (2019). LC-MS/MS-based separation and quantification of Marfey’s reagent derivatized proteinogenic amino acid DL-stereoisomers. J. Amer. Soc. Mass Spectrom..

[32] Ikuina Y., Bando C., Yoshida M., Yano H., Saitoh Y. (1997). MS-681a, b, c and d, new inhibitors of myosin light chain kinase from *Myrothecium* sp. II. Physico-chemical properties and structure elucidation. J. Antibiotics.

[33] Christner C., Zerlin M., Gräfe U., Heinze S., Küllertz G., Fischer G. (1997). Lipohexin, a new inhibitor of prolyl endopeptidase from *Moeszia lindtneri* (HKI-0054) and *Paecilomyces* sp. (HKI-0055; HKI-0096). II. Inhibitory activities and specificity. J. Antibiotics.

[34] Heinze S., Ritzau M., Ihn W., Hülsmann H., Schlegel B., Dornberger K., Fleck W.F., Zerlin M., Christner C., Gräfe U. (1997). Lipohexin, a new inhibitor of prolyl endopeptidase from *Moeszia lindtneri* (HKI-0054) and *Paecilomyces* sp. (HKI-0055; HKI-0096). I. Screening, isolation and structure elucidation. J. Antibiotics.

[35] Hegde V.R., Silver J., Patel M., Gullo V.P., Puar M.S., Das P.R., Loebenberg D. (2003). Novel fungal metabolites as cell wall active antifungals: Fermentation, isolation, physico-chemical properties, structure and biological activity. J. Antibiotics.

[36] Stump H., Stahl W., Wink J., Markus A., Kogler H., Backhaus J. (2001). Antifungal Peptides from Scleroderma Texense. U.S. Patent.

[37] Pierson N.A., Chen L., Russell D.H., Clemmer D.E. (2013). *Cis–Trans* isomerizations of proline residues are key to bradykinin conformations. J. Amer. Chem. Soc..

[38] Glover M.S., Shi L., Fuller D.R., Arnold R.J., Radivojac P., Clemmer D.E. (2015). On the split personality of penultimate proline. J. Amer. Soc. Mass Spectrom..

[39] Siebler C., Maryasin B., Kuemin M., Erdmann R.S., Rigling C., Grünenfelder C., Ochsenfeld C., Wennemers H. (2015). Importance of dipole moments and ambient polarity for the conformation of Xaa–Pro moieties—A combined experimental and theoretical study. Chem. Sci..

[40] Loureiro A., Pais C., Sampaio P. (2019). Relevance of macrophage extracellular traps in *C. albicans* killing. Front. Immunol..

[41] Song X.-Y., Xie B.-B., Chen X.-L., Zhang Y.-Z., Zeilinger S., Martín J.-F., García-Estrada C. (2015). Biosynthesis and molecular genetics of peptaibiotics—Fungal peptides containing alpha, alpha-dialkyl amino acids. Biosynthesis and Molecular Genetics of Fungal Secondary Metabolites.

[42] Toniolo C., Crisma M., Formaggio F., Peggion C., Epand R.F., Epand R.M. (2001). Lipopeptaibols, a novel family of membrane active, antimicrobial peptides. Cell. Mol. Life Sci..

[43] Valdés-Santiago L., Ruiz-Herrera J. (2014). Stress and polyamine metabolism in fungi. Front. Chem..

