# Supplementary material for: Sphaerostilbellins, New Antimicrobial Aminolipopeptide Peptaibiotics from Sphaerostilbella toxica
Source: Biomolecules. 2020 Sep 26;10(10):1371. doi: 10.3390/biom10101371 (PMC7600149; doi:10.3390/biom10101371)
Supplement: Supplementary file 1 [file biomolecules-10-01371-s001.pdf]

Supplementary Data

Sphaerostilbellins, new antimicrobial aminolipopeptides from *Sphaerostilbella toxica*

Bruno Perlatti [1], Connie B. Nichols [2], J. Andrew Alspaugh [2], James B. Gloer [3], Gerald F. Bills [1,*]

[1] Texas Therapeutics Institute, The Brown Foundation Institute of Molecular Medicine, University of Texas Health Science Center at Houston, Houston, Texas 77054, USA

[2] Departments of Medicine and Molecular Genetics & Microbiology, Duke University Medical Center, Durham, NC 27710, USA

[3] Department of Chemistry, University of Iowa, Iowa City, Iowa 52242, USA

*Author to whom correspondence should be addressed.

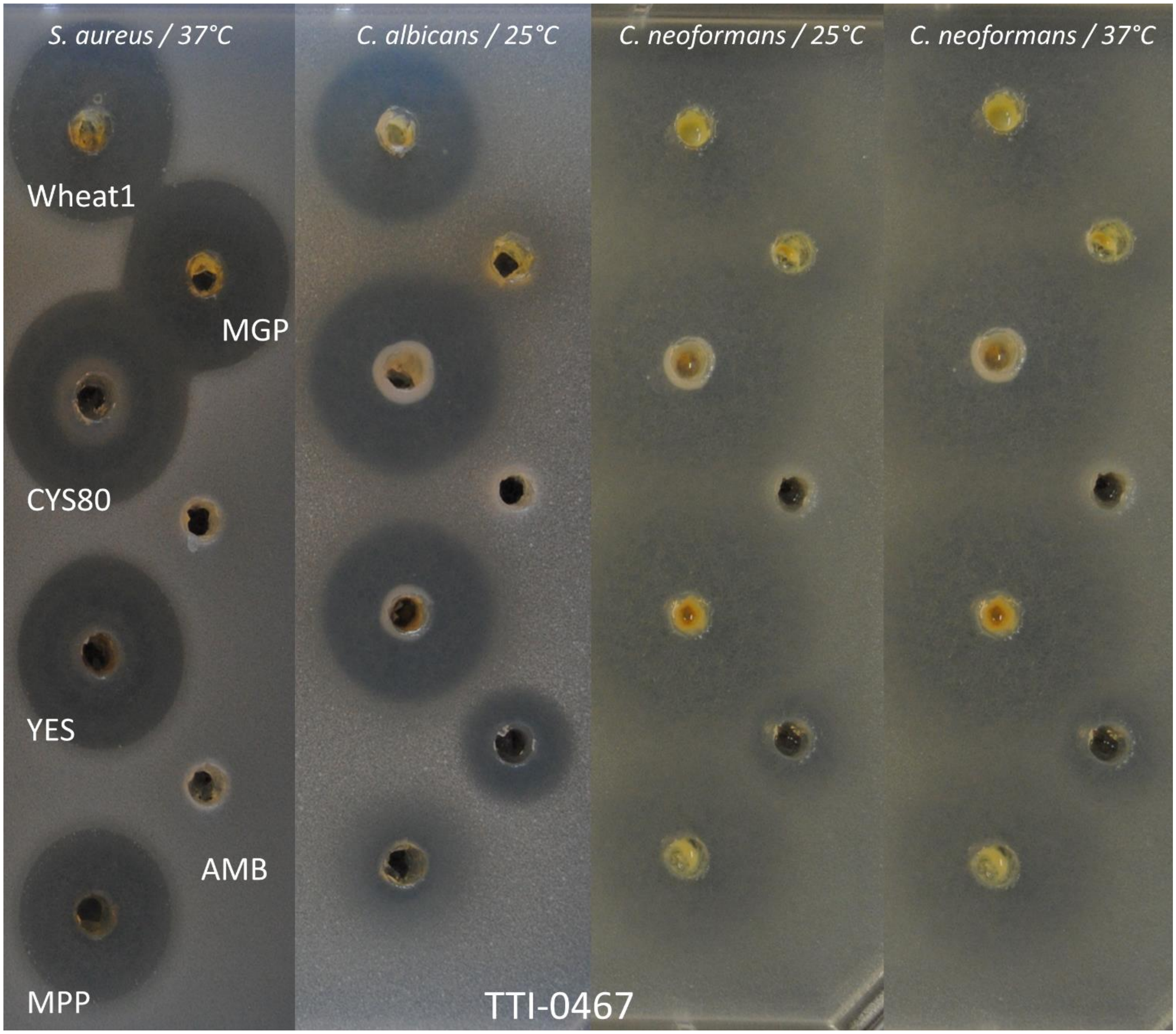

Figure S1. Zone of inhibition assay of extracts from strain TTI-0467 cultured in five different media against *C. neoformans* at 37 °C. See Methods for media formulations.

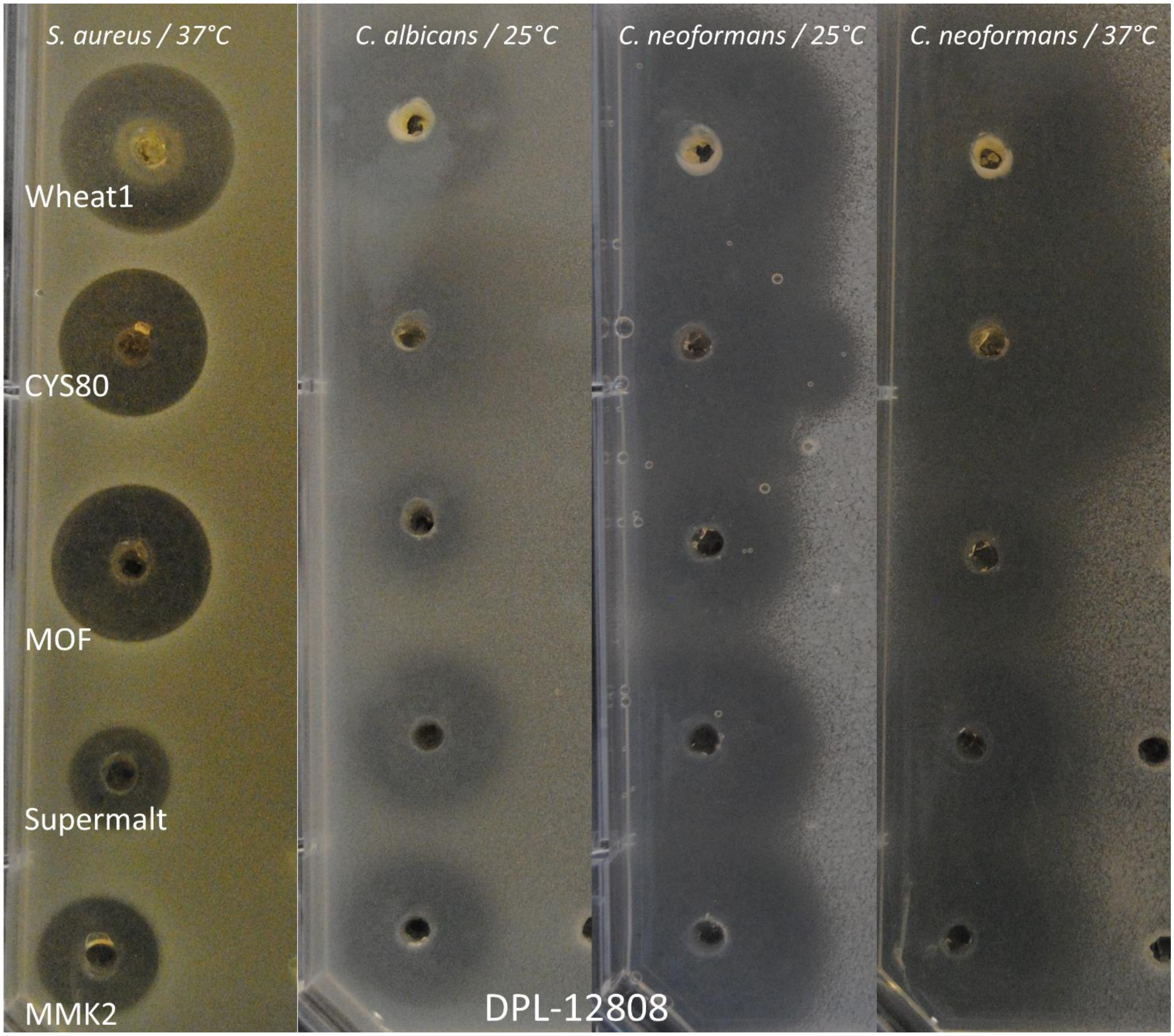

Figure S2. Zone of inhibition assay of extracts from strain DPL-12808 cultured in five different media against *C. neoformans* at 37 °C. See Methods for media formulations.

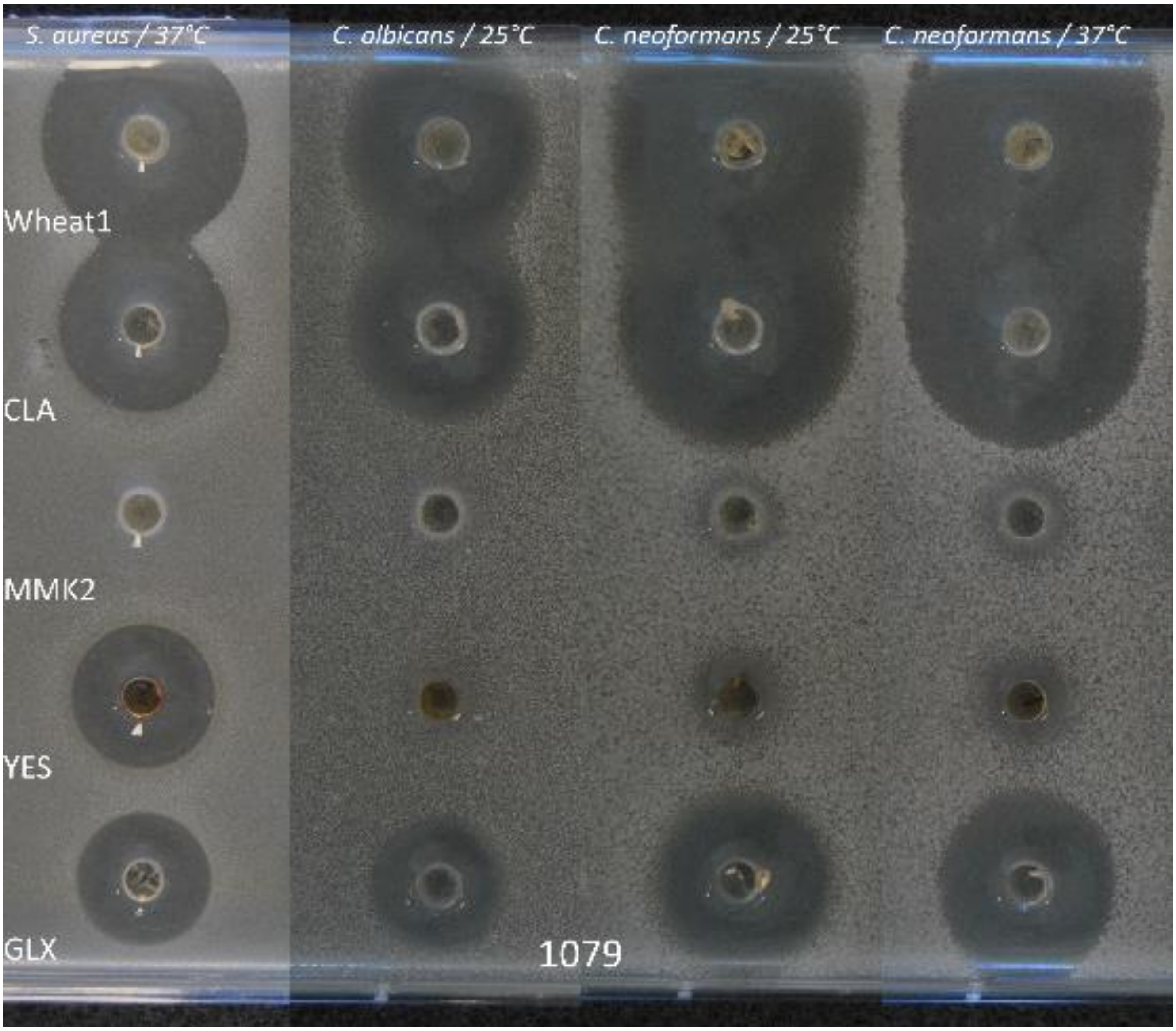

Figure S3. Zone of inhibition assay of extracts of strain TTI-1079 cultured in five different media against *C. neoformans* at 37 °C. See Methods for media formulations.

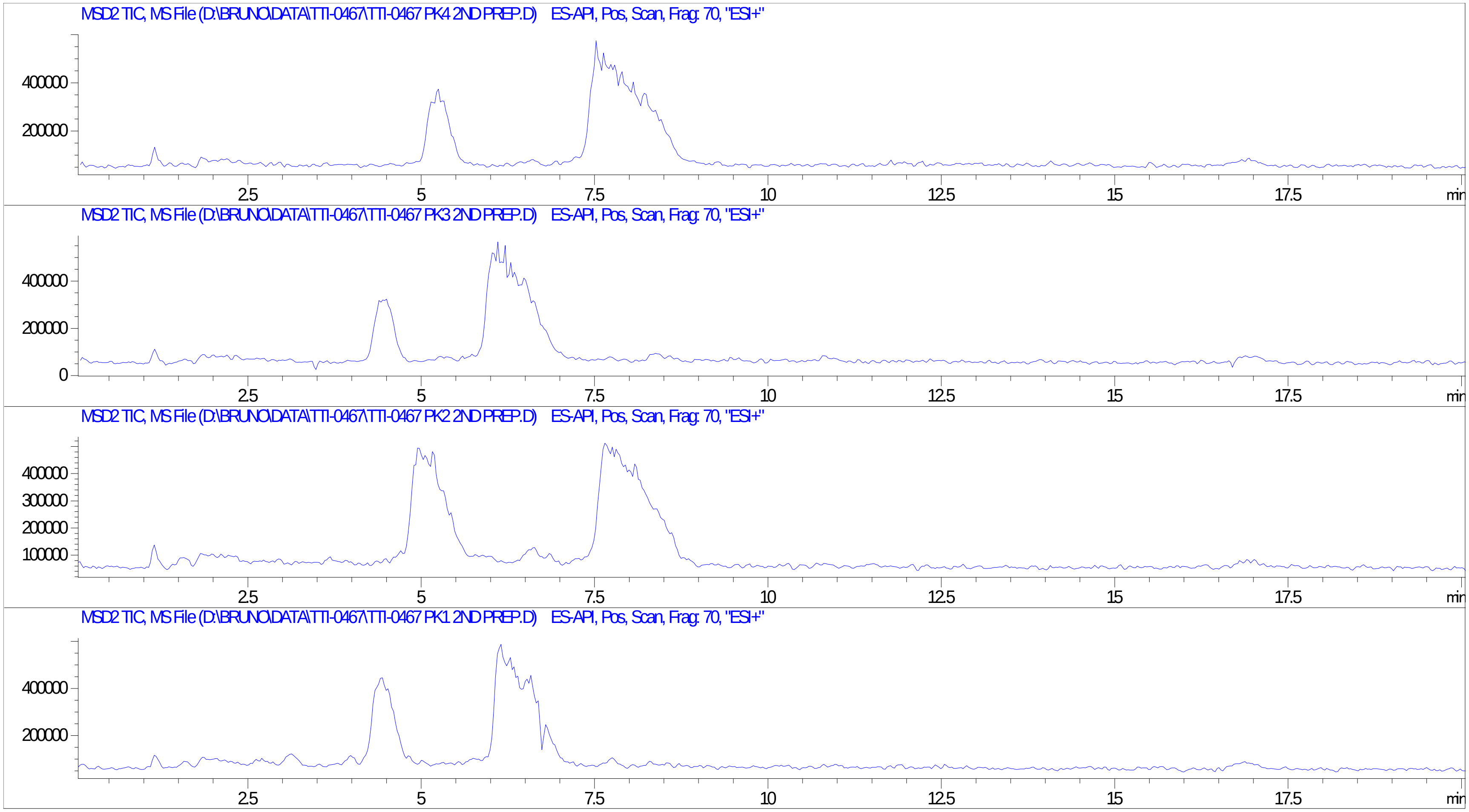

Figure S4. LC-MS of peaks 1-4 after semi-preparative HPLC isolation, highlighting the interconversion of peaks 1-3 and 2-4.

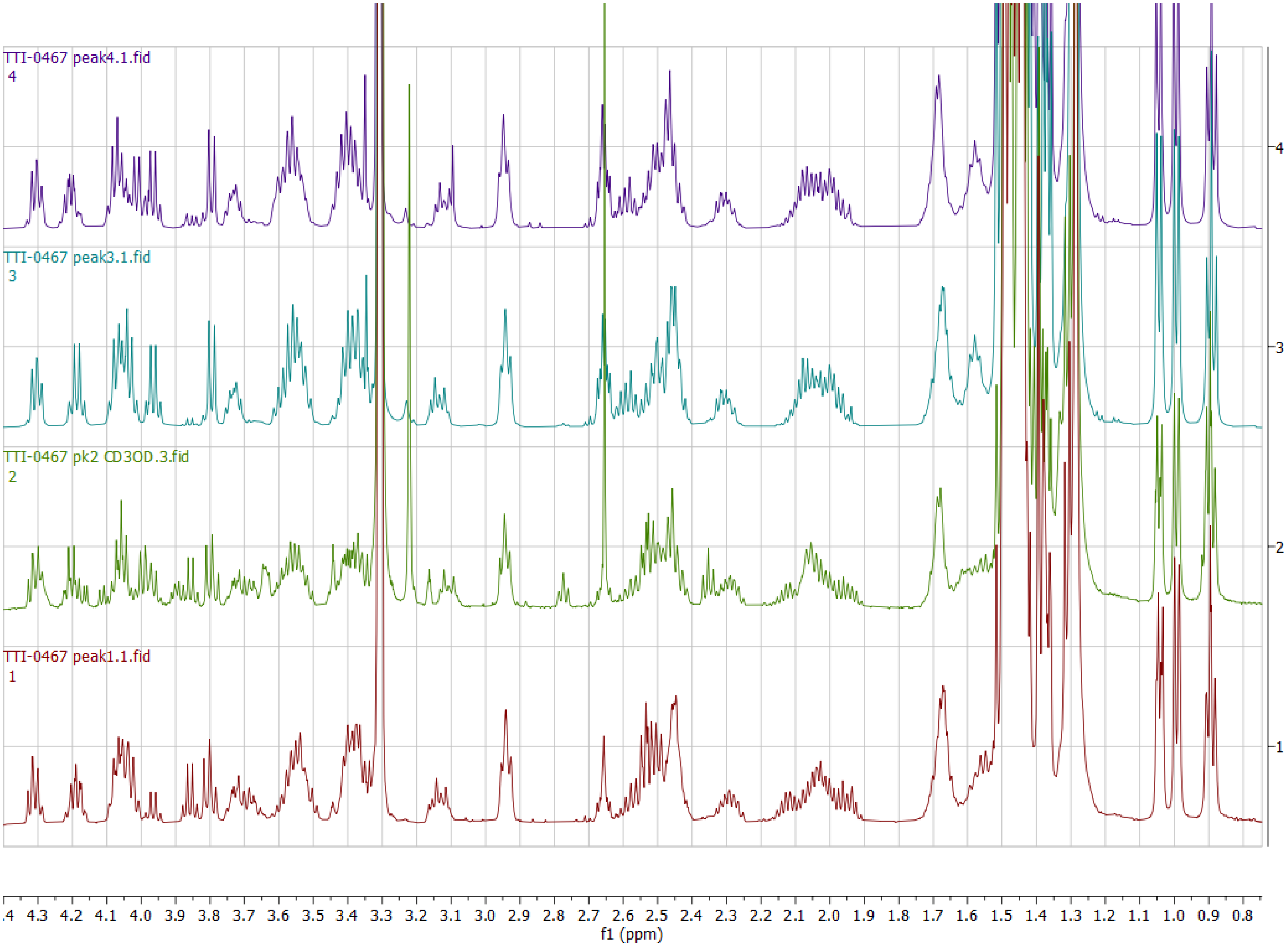

Figure S5. ^{1}H NMR spectrum of peaks 1-4 (500 MHz, CD_3OD).

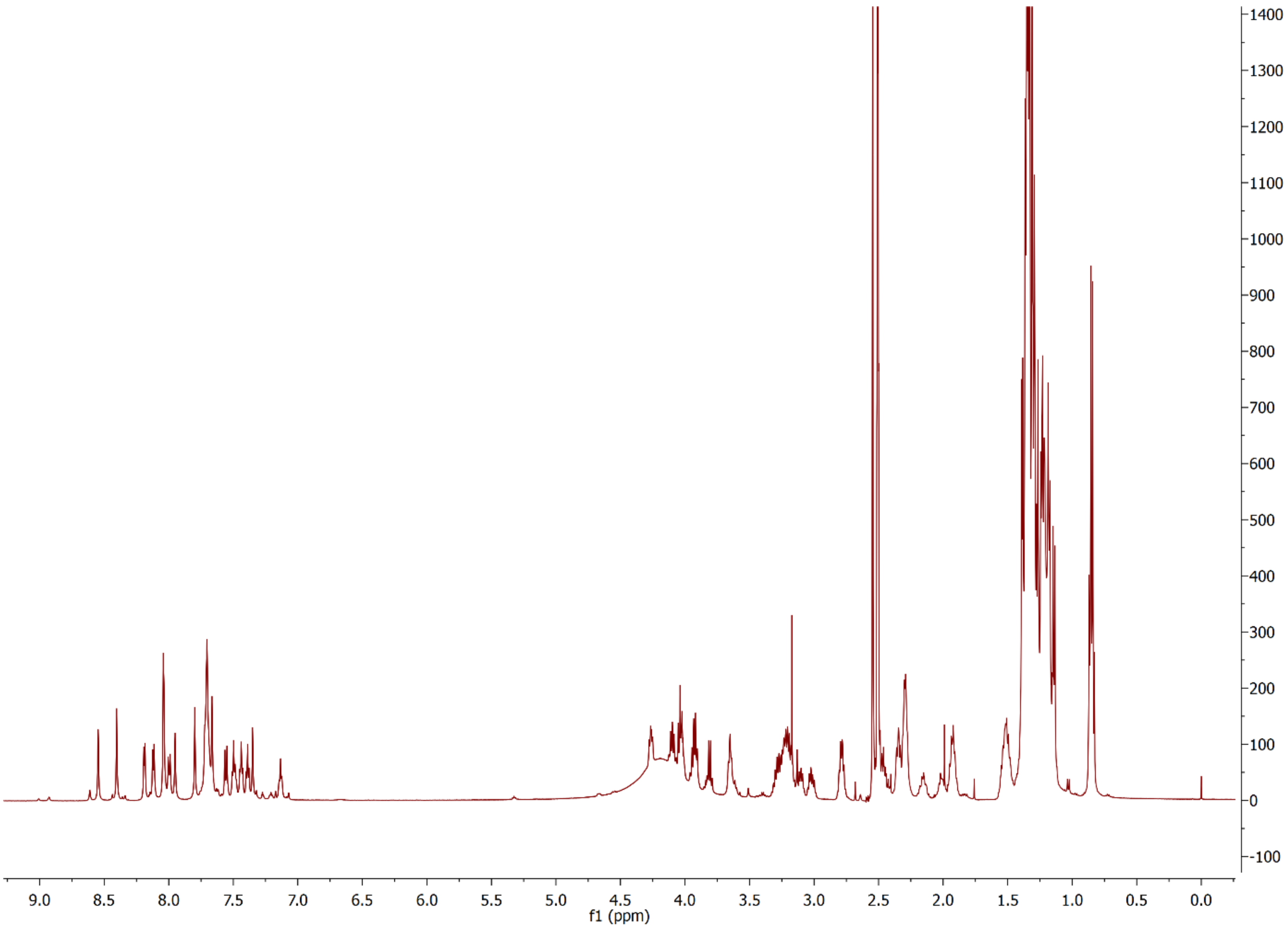

Figure S6. ^{1}H NMR spectrum of **1** (500 MHz, DMSO-*d*$_6$).

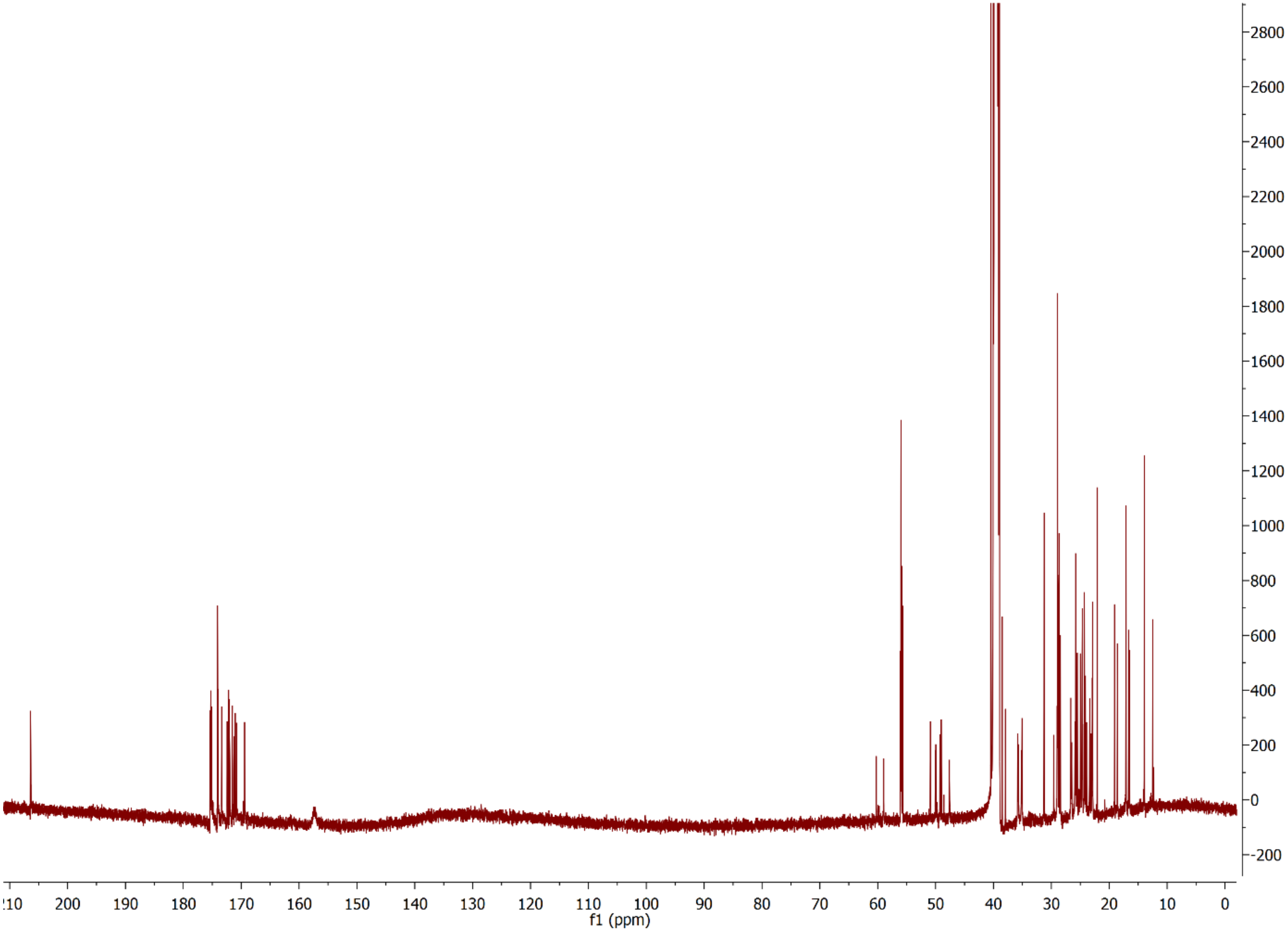

Figure S7. ^{13}C NMR spectrum of **1** (125 MHz, DMSO-d_6).

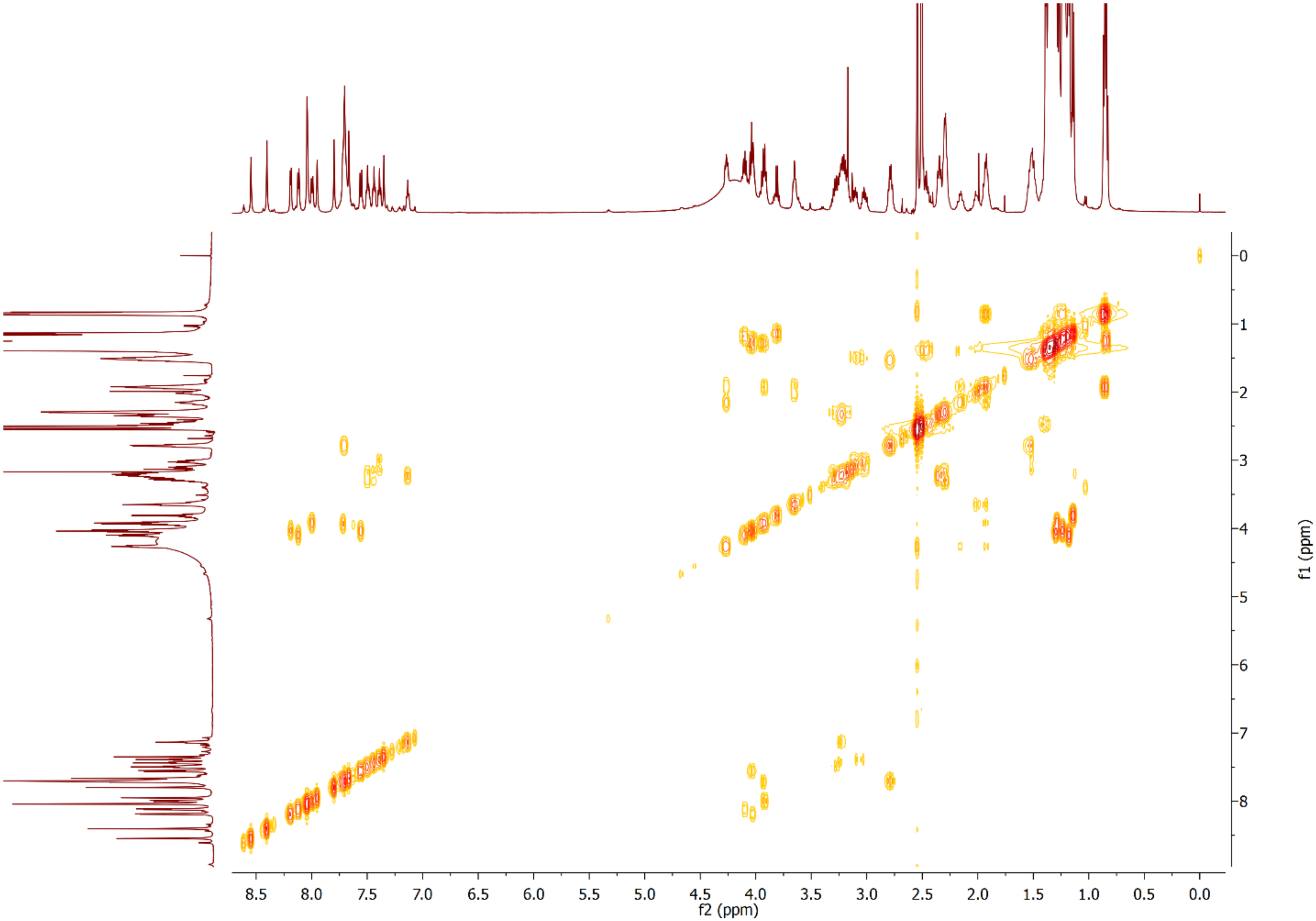

Figure S8. COSY spectrum of **1** (500 MHz, DMSO-d_6).

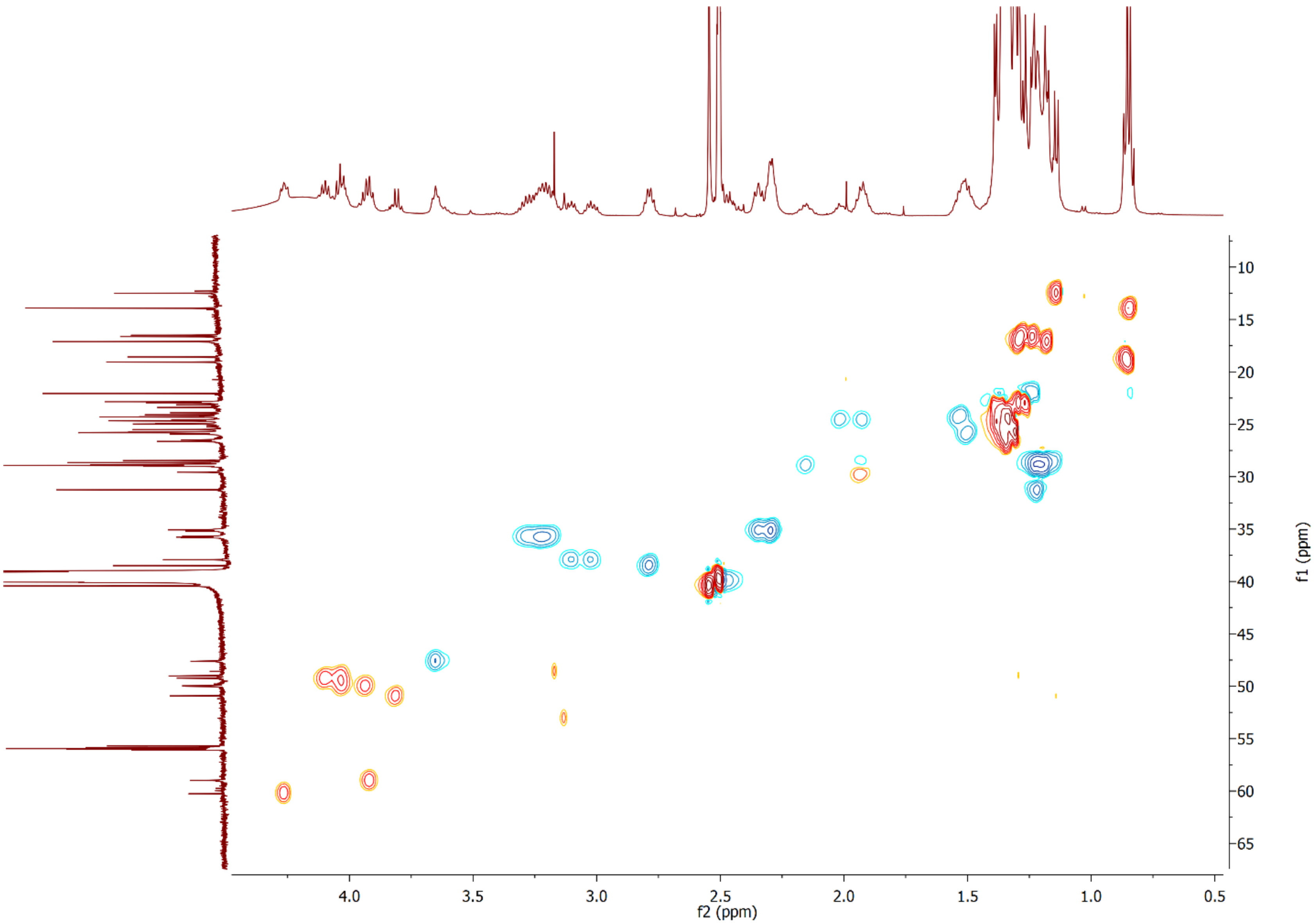

Figure S9. HSQC spectrum of **1** (500 MHz, DMSO-*d*6).

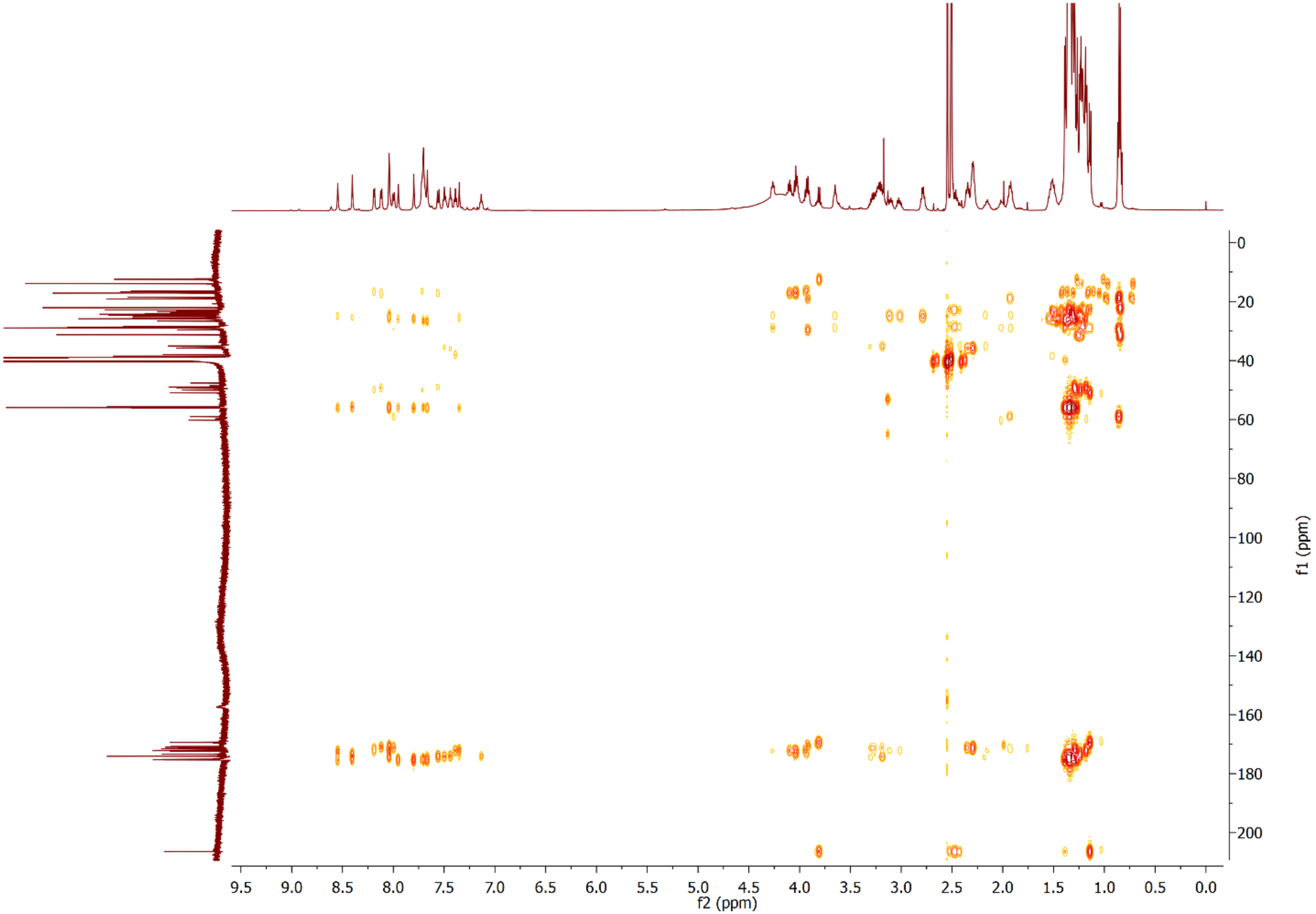

Figure S10. HMBC spectrum of **1** (500 MHz, DMSO-d_6).

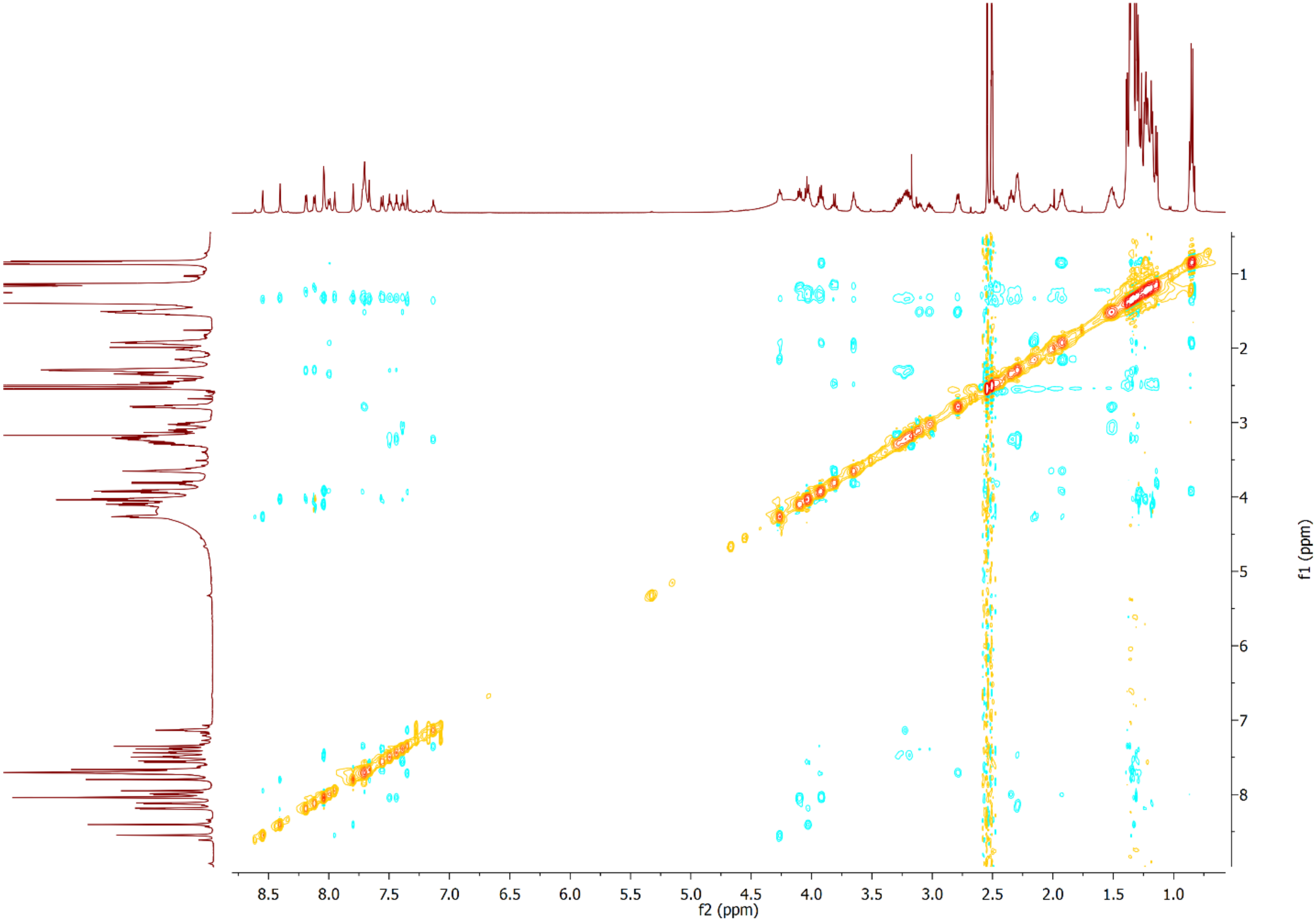

Figure S11. ROESY spectrum of **1** (500 MHz, DMSO-d_6).

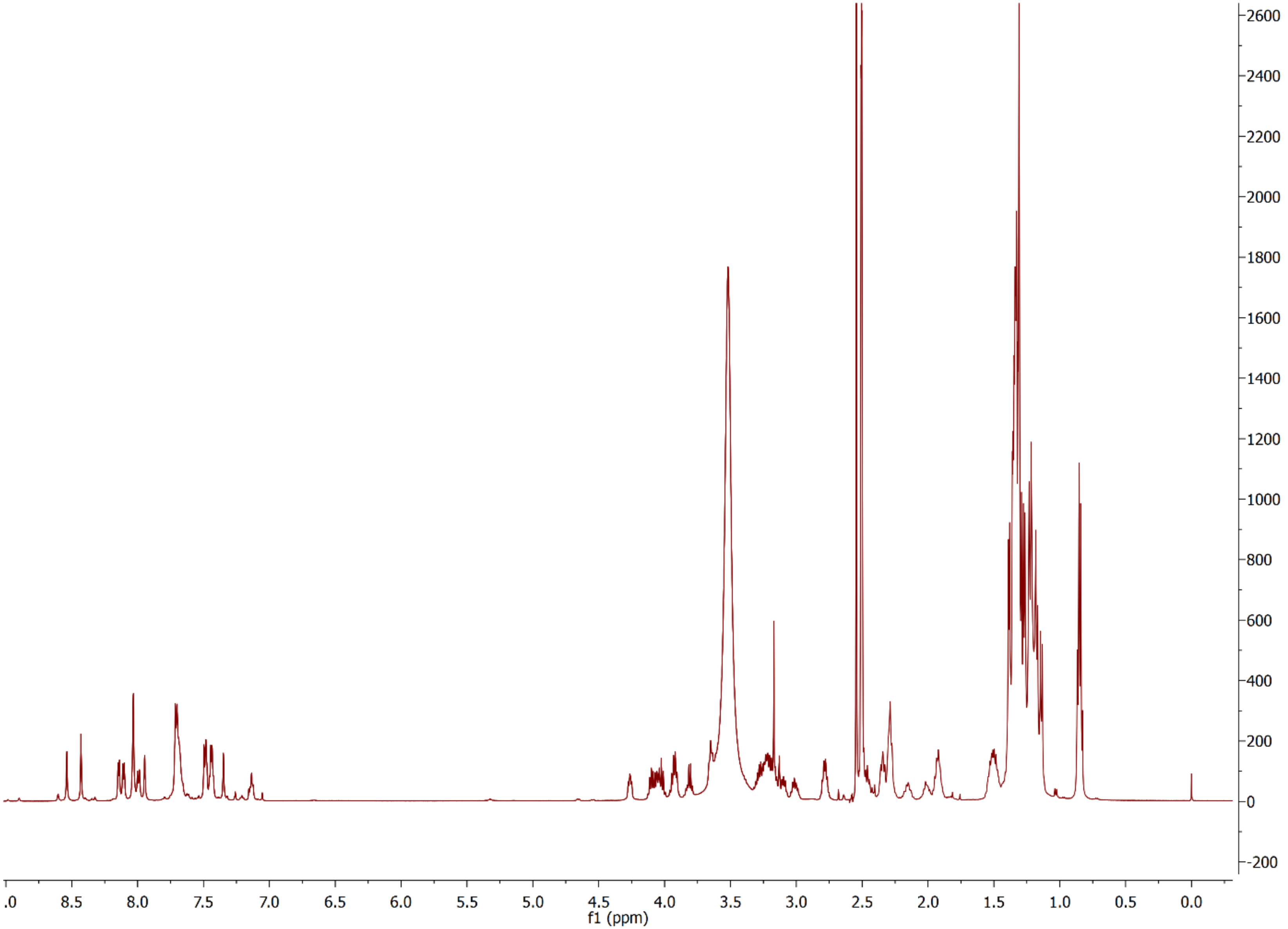

Figure S12. ^{1}H NMR spectrum of **2** (500 MHz, DMSO-d_6).

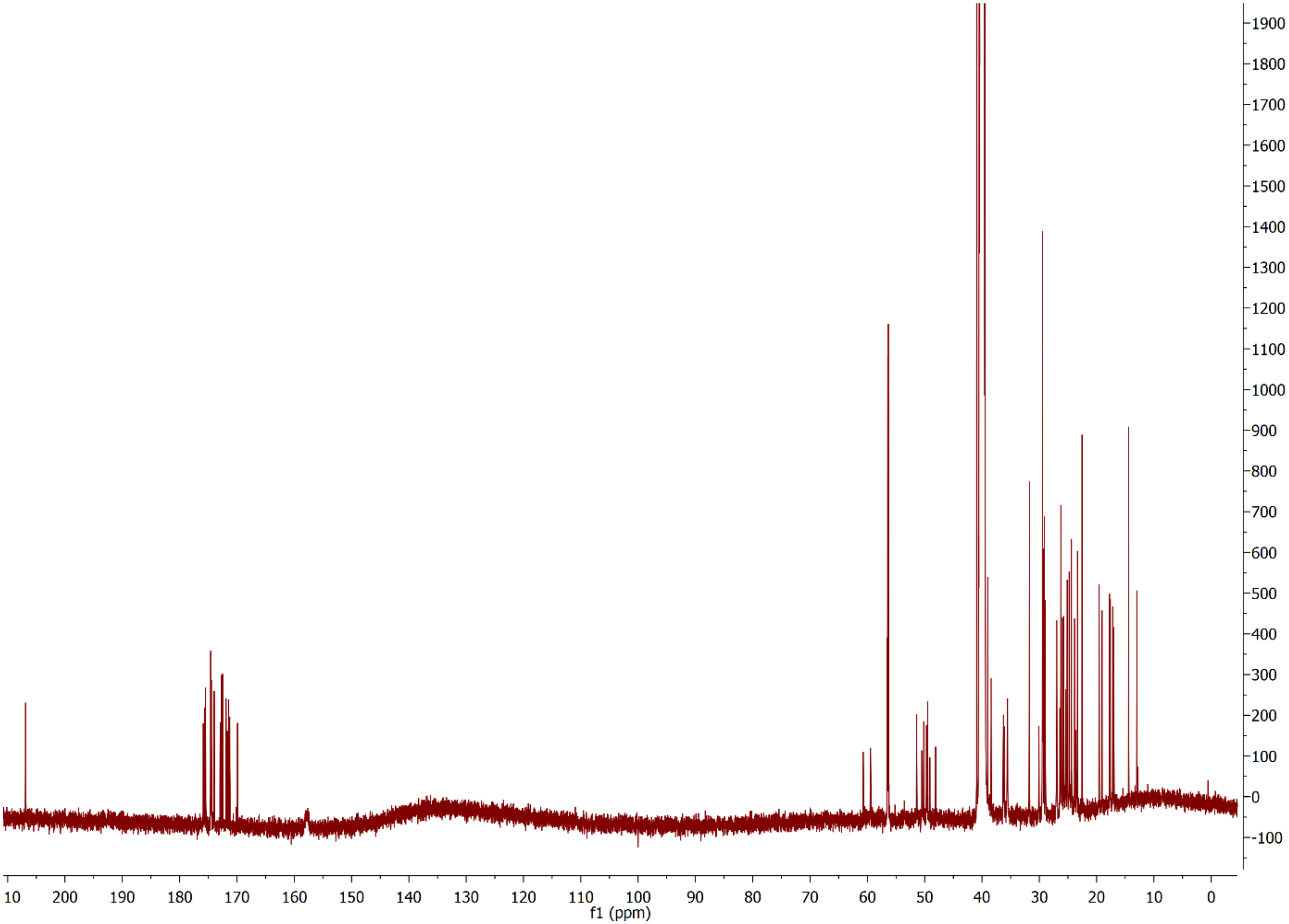

Figure S13. ^{13}C NMR spectrum of **2** (125 MHz, DMSO-d_6).

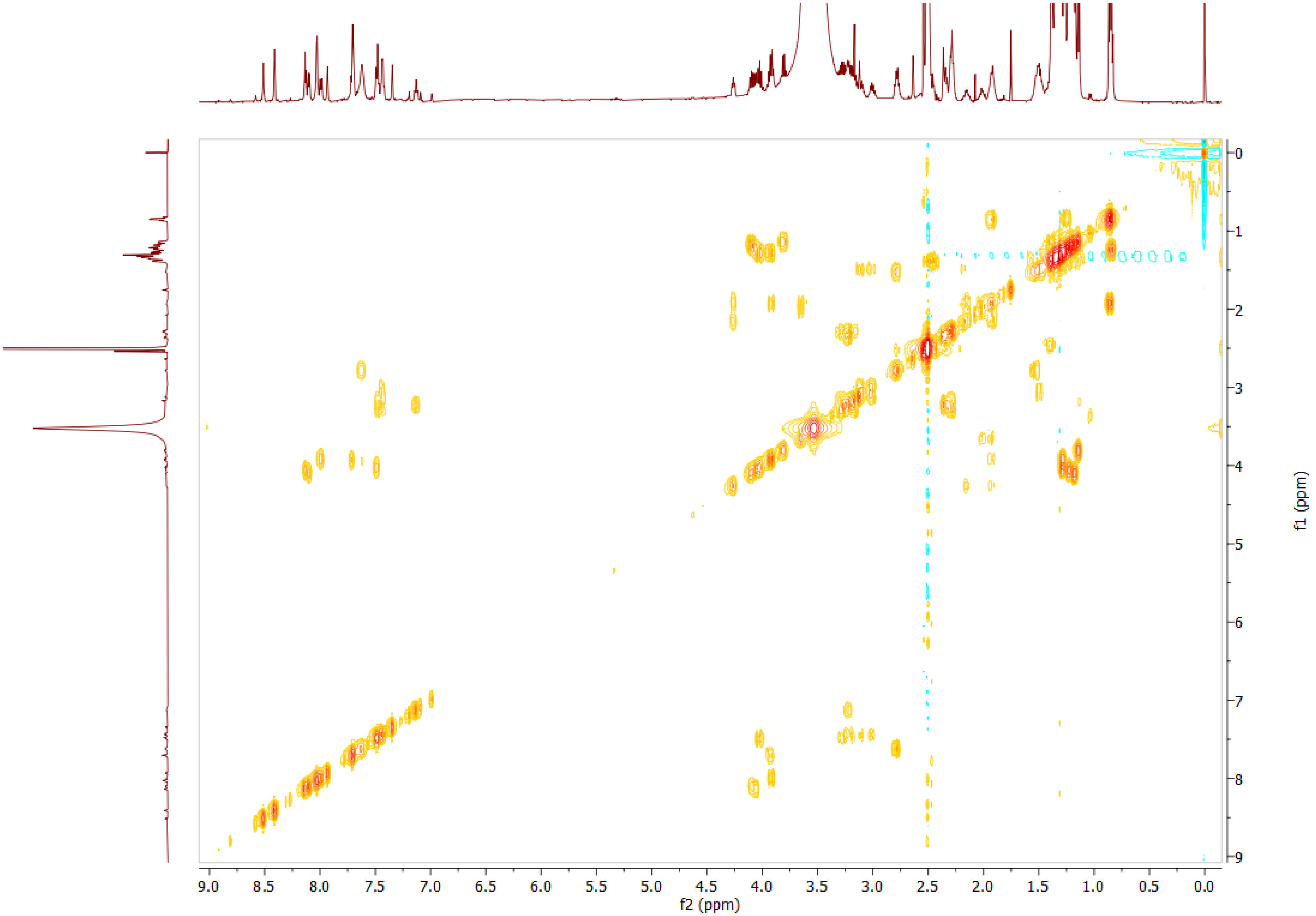

Figure S14. COSY spectrum of **2** (500 MHz, DMSO-d_6).

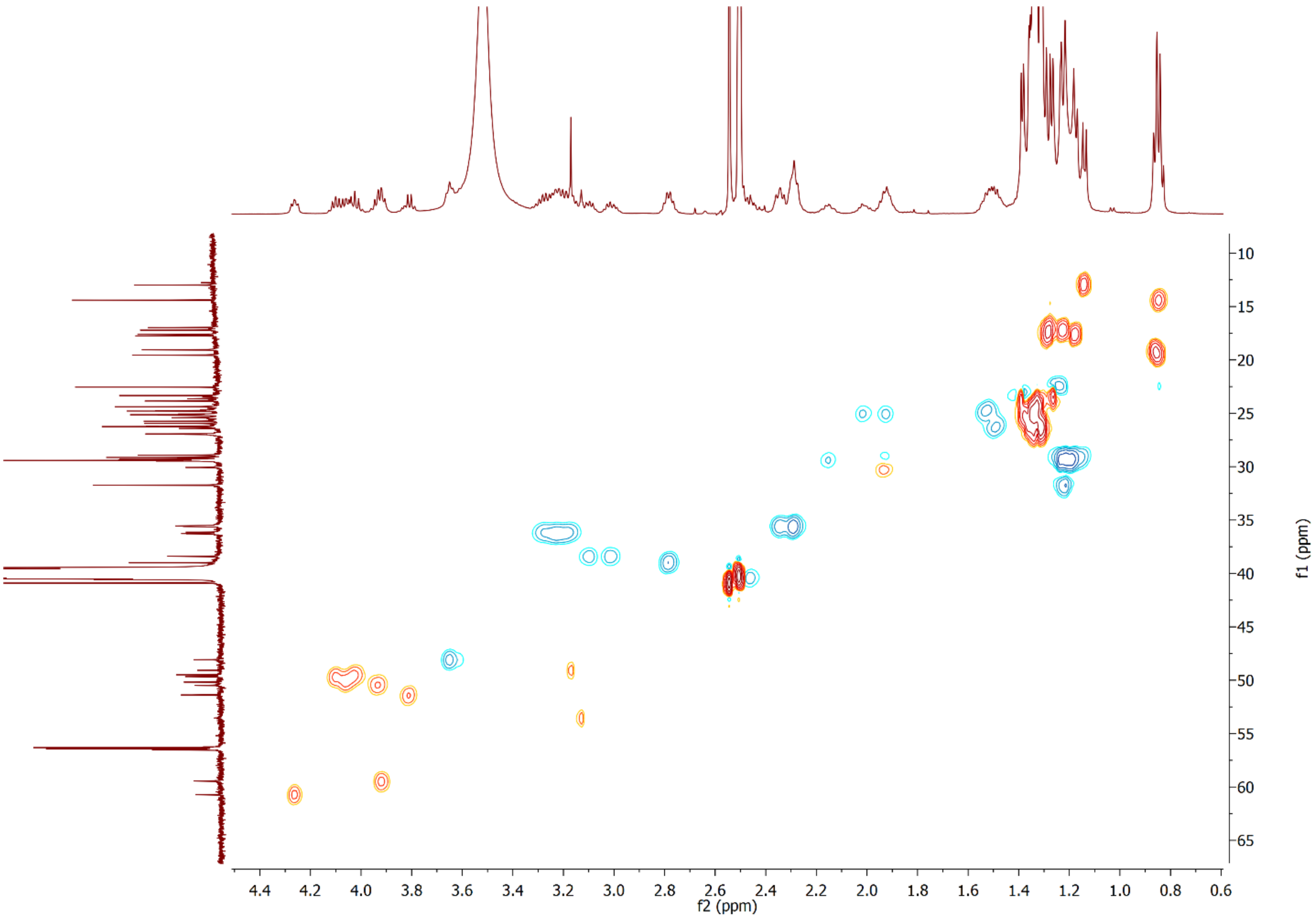

Figure S15. HSQC spectrum of **2** (500 MHz, DMSO-d_6).

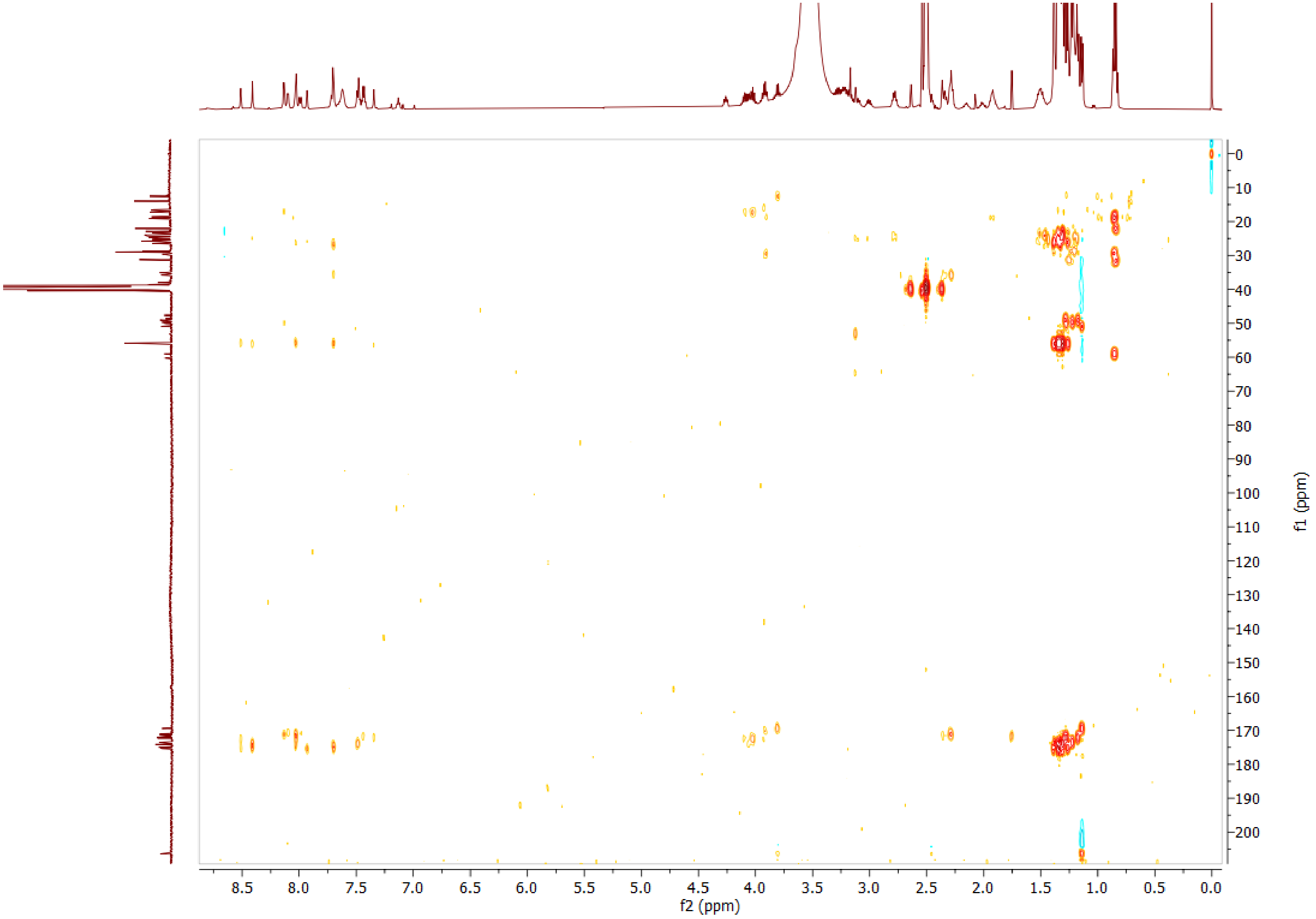

Figure S16. HMBC spectrum of **2** (500 MHz, DMSO-d_6).

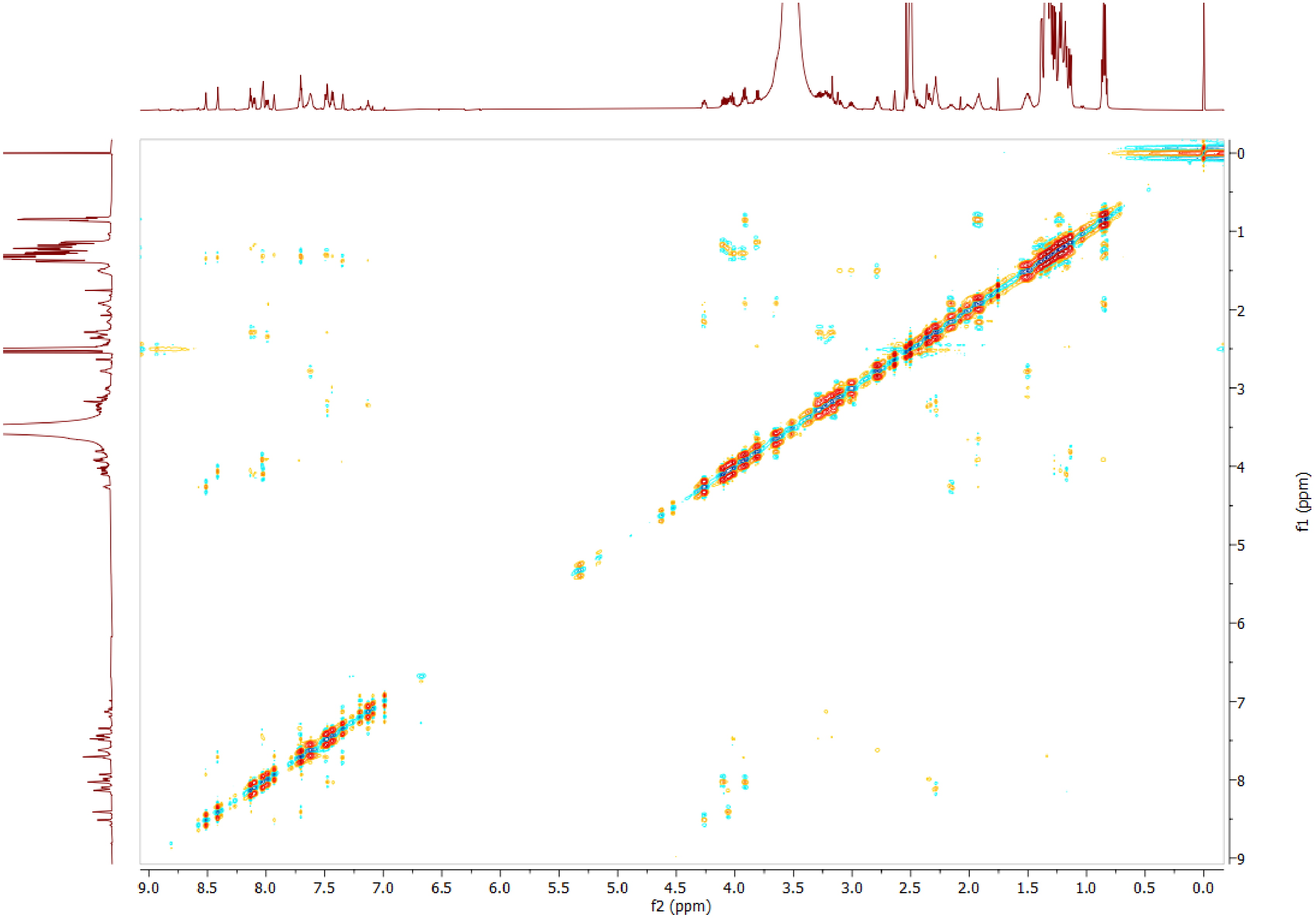

Figure S17. ROESY spectrum of **2** (500 MHz, DMSO-d_6).

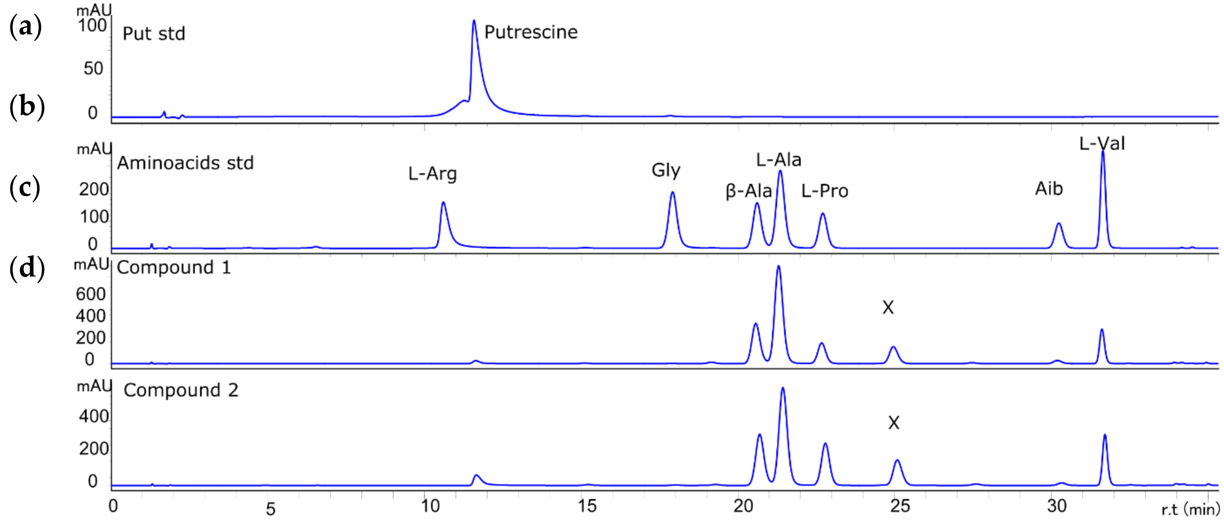

Figure S18. LCMS chromatograms obtained after sample derivatization with Marfey's reagent. a) Putrescine standard; b) Amino acid standards; c) Compound **1** hydrolyzate; d) Compound **2** hydrolyzate. X represents a side-product.

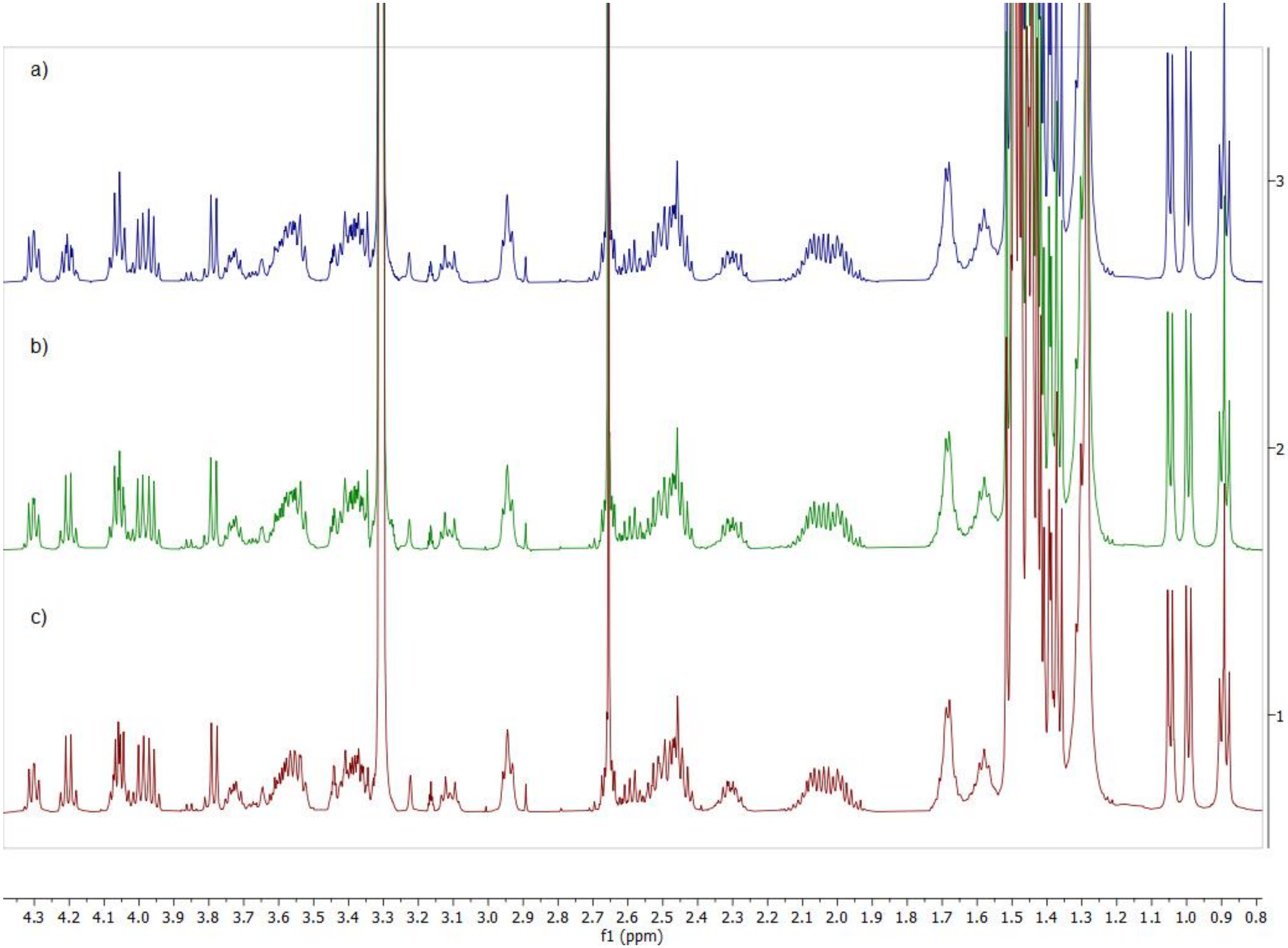

Figure S19. ^{1}H NMR (500 MHz, CD_3OD) spectra of sphaerostilbellin A at different times. a) 0 days; b) 4 days; c) 110 days.
